# Synergistic encapsulation of thymol in porphyrin-based porous frameworks enhances the multifunctionality of chitosan/polyvinyl alcohol films for food preservation

**DOI:** 10.1016/j.crfs.2026.101605

**Published:** 2026-10-20

**Authors:** Huajuan Wang, Mengni Zhou, Yeting Xu, Yan Huang, Liang Ke, Wei Xu, Yang Yi, Min Zhou

**Affiliations:** a Hubei Key Laboratory for Processing and Transformation of Agricultural Products, College of Food Science and Engineering, Wuhan Polytechnic University, Wuhan, 430023, China; b Huangshi Central Hospital, Huangshi, 435000, China; c Hubei Huangshan Industry Group Company Limited, Xiantao, 433022, China; d Hubei Huangshan Industry Technology Research Institute Company Limited, Xiantao, 433022, China

**Keywords:** Synergistic antibacterial, Porphyrinic framework, Chitosan/ polyvinyl alcohol, Active packaging, Food preservation

## Abstract

Significant losses from food perishability and the ecological burden of petroleum-based packaging demand innovative bio-based active solutions. Herein, we report a multifunctional chitosan/polyvinyl alcohol film incorporated with thymol-loaded porphyrinic metal-organic framework (MOF). This film is denoted as CS/PVA/PT, where PT represents PCN@Thy (i.e., PCN-224 loaded with thymol). Addressing the limitations of single-mode packaging, the synergistic encapsulation of thymol within PCN-224 acts as a dual-function booster: its porous structure ensures the sustained release of thymol, while its photodynamic effect drives continuous reactive oxygen species (ROS) generation, establishing a robust synergistic antibacterial mechanism. The optimized CS/PVA/PT film incorporating 1.2 wt% PT (relative to the total mass of CS/PVA at 1:1, w/w) exhibited superior mechanical and barrier properties, alongside potent activity against bacteria (*P. aeruginosa*, *A. hydrophila*, *V. parahaemolyticus*) and fungi (*Botrytis cinerea*). Practically, the film effectively extended the shelf life of fish slices and strawberries. Importantly, the Zirconium (Zr) migration level (0.036 mg/kg) was far below the permissible limits (2 mg/kg) set by the European Directorate for the Quality of Medicines & HealthCare (EDQM), confirming food contact safety. Consequently, this film holds great promise as an innovative and reliable solution for extending shelf life and mitigating food waste.

## Highlights

• Synergistic encapsulation of thymol in PCN-224 yields multifunctional CS/PVA films.
• Incorporating PCN@Thy enhanced the mechanical, barrier, and antioxidant properties.
• Sustained thymol release and photodynamic ROS drove robust antimicrobial activity.
• The films effectively extended the shelf life of both fish slices and strawberries.

## Graphical abstract

**Figure:**
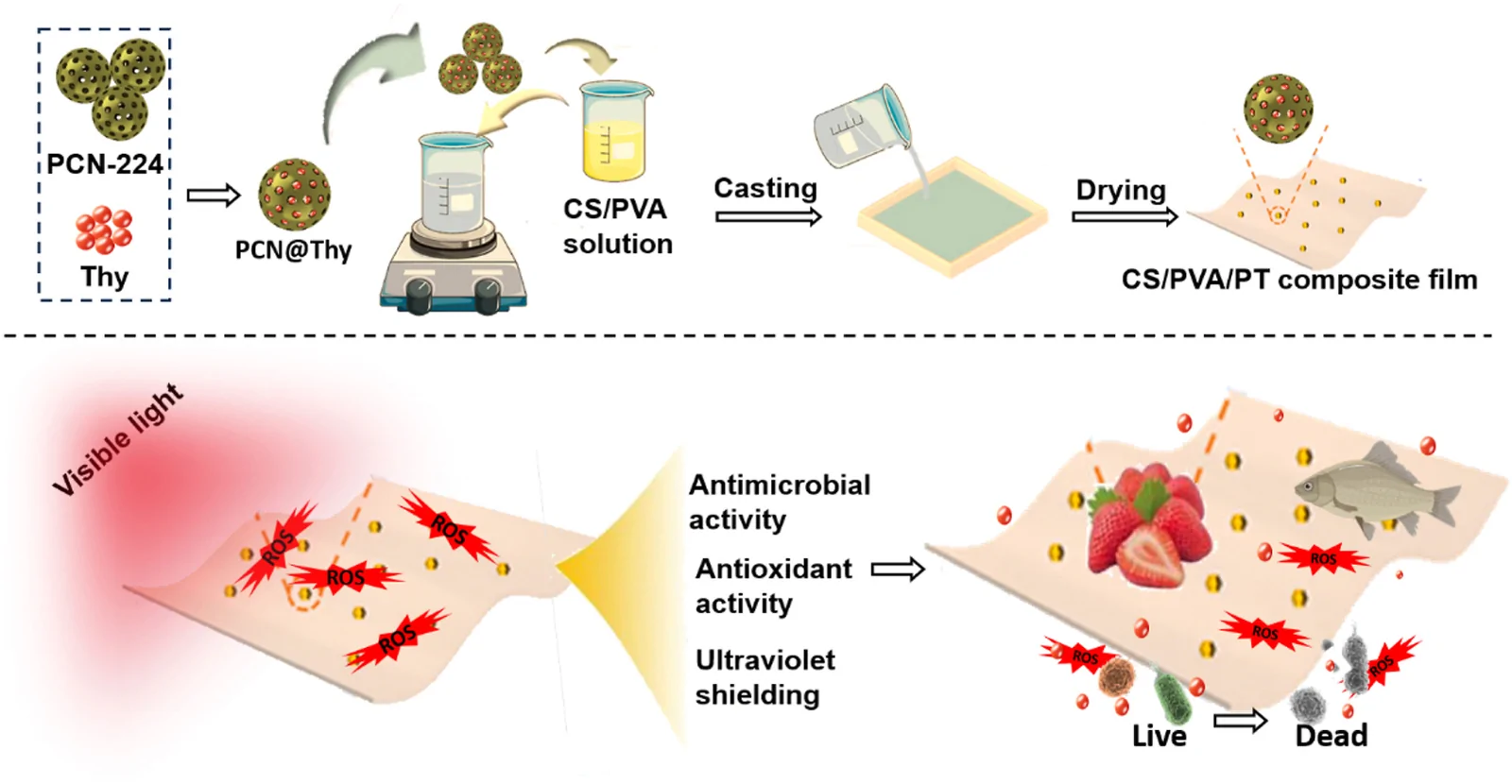

A multifunctional CS/PVA/PT film was fabricated for food preservation, achieving robust synergistic antibacterial activity via photodynamic ROS generation and sustained thymol release.

## 1 Introduction

Approximately one-third of global food production is lost prior to consumption, primarily due to spoilage along the supply chain (Y＀＀X. Liu et al., 2025a). This not only inflicts substantial economic losses but also poses a significant threat to global food security (Hassoun et al., 2023). Among various mitigation strategies, packaging plays a crucial role in maintaining food quality. Not only can it effectively shield food from external microbial contamination and environmental factors (e.g., light) to extend shelf life, but it also retains its sensory and nutritional profile (D'Almeida and de Albuquerque, 2024). Conventional food packaging relies heavily on petroleum-based plastics, such as polyethylene (PE) and polypropylene (PP), which are widely used owing to their robust barrier properties, low cost and manufacturing adaptability (X＀＀Y. Liu et al., 2025b). However, these materials degrade exceptionally slowly in the natural environment (S. Zhang et al., 2025a); for instance, PE films may require 300 to 400 years to decompose completely (Oluwoye et al., 2023). This persistence not only causes severe environmental pollution but also leads to the continuous accumulation of microplastics in the food chain, ultimately posing a major threat to public health (Yibing Zhang et al., 2025c). Furthermore, conventional packaging is largely restricted to passive barrier properties, lacking active functions such as antimicrobial and antioxidant capabilities. Such packaging fails to provide sustained preservation, leaving the issue of food spoilage unresolved. Coupled with the growing consumer demand for sustainable lifestyles, conventional petroleum-based packaging is becoming increasingly inadequate for modern food preservation needs (Shen et al., 2020). Consequently, developing novel packaging materials that are green, biodegradable, and multifunctional has become imperative.

Recently, driven by the growing demand for sustainable materials, biopolymer-based packaging has emerged as a major research focus. Common film-forming biopolymers primarily include polysaccharides and proteins (Zhu et al., 2026). These materials are not only biodegradable and edible but also effectively eliminate the risk of microplastic residue on food. Among them, natural polysaccharides such as chitosan (CS) (Flórez et al., 2022), cellulose (Zhao et al., 2025) and starch (Basdeki et al., 2026) are highly favored due to their abundance, renewability, and excellent biocompatibility. Specifically, CS has garnered widespread application as a foundational film matrix owing to its superior film-forming capability and economic viability. Moreover, polyvinyl alcohol (PVA) has emerged as a highly desirable film-forming material due to its non-toxicity, biodegradability, and exceptional film-forming ability (Yang et al., 2023). Nevertheless, films based on a single biopolymer often exhibit inherent defects; for instance, pure CS films suffer from inadequate water resistance (Wen et al., 2021), whereas PVA films show suboptimal mechanical and thermal stability (Zhang et al., 2024). Given their high compatibility, and the ability of PVA to disrupt the regularity of chitosan molecular chains and reduce its crystallinity (Habiba et al., 2017), compounding CS with PVA has emerged as an effective strategy to enhance the comprehensive physical properties of the films (Salleh et al., 2025). However, despite the improved basic physical properties of chitosan/polyvinyl alcohol (CS/PVA) composite films, they still lack intrinsic antibacterial and antioxidant activities, which severely restricts their application in extending food shelf life. To endow these films with active preservation capabilities, researchers often introduce natural active substances into the film-forming system (Zhang et al., 2024). Particularly, the integration of plant essential oils with nanocarriers has attracted significant attention. This system not only resolves the volatility and poor water solubility of essential oils via nano-encapsulation, but also achieves long-lasting and broad-spectrum antibacterial effects through synergistic interactions between the essential oils and the nanocarriers, providing a promising avenue for the development of highly efficient bio-based active packaging.

Thymol (Thy) is a natural monoterpene phenolic compound that exhibits excellent antibacterial and antioxidant activities, as well as good biosafety (Munteanu and Vasile, 2021). Nevertheless, its inherent drawbacks, such as poor water solubility and easy volatility, limit its direct application in food packaging systems (Li et al., 2025). To circumvent these problems, Thy is often encapsulated within various delivery vehicles, such as nanoparticles (Yun Zhang et al., 2025b) and emulsions (Phyo et al., 2024), thereby effectively enhancing its stability and enabling sustained release.

Porphyrin-based porous coordination network (PCN-224) is a porphyrin-based zirconium (Zr) metal–organic framework (MOF) in the nanoscale range, designed so that the porphyrin units act as built-in photosensitizers able to generate reactive oxygen species (ROS, mainly singlet oxygen) under light irradiation (Zhang et al., 2021). Characterized by high specific surface area, excellent water solubility, and good biocompatibility, it has been widely utilized in drug delivery and photodynamic therapy (Wang et al., 2023a). Recently, its potential as a nanocarrier for natural active ingredients has gained increasing attention. When integrated into active food packaging, PCN-224 confers a distinctive dual advantage: its highly porous networks not only enables high-capacity loading and sustained release of antibacterial agents, but also inherently acts as a potent photosensitizer. Upon excitation by light of specific wavelengths, its porphyrin structure acts as a photosensitizer, converting molecular oxygen into singlet oxygen (^1^O_2_) and other ROS (e.g., ·OH and H_2_O_2_) via energy or electron transfer processes, which induce fatal oxidative stress in bacteria by disrupting essential biomacromolecules such as proteins and DNA (Wang et al., 2023b). This synergistic mode of physical encapsulation and photodynamic inactivation provides a highly promising strategy for developing advanced active packaging.

In this study, thymol was innovatively loaded into PCN-224 (PCN@Thy) to simultaneously achieve the controlled release of the loaded thymol via the high specific surface area of PCN-224 and generate ROS through its photodynamic action. This dual functionality enables a synergistic enhancement of chemical inhibition and photodynamic sterilization. Subsequently, the PCN@Thy nanocomposites were incorporated into a biodegradable CS/PVA matrix to fabricate a novel multifunctional active packaging film (CS/PVA/PT, where PT refers to PCN@Thy incorporated in the film) (Scheme 1). The mechanical properties, as well as functional activities (antibacterial and antifungal activity) and biosafety (evaluated by hemolysis, MTT cytotoxicity, and Zr migration assays), were systematically evaluated, alongside their practical preservation efficacy on perishable foods. This work aims to overcome the limitations of conventional single-function and non-degradable packaging, providing important theoretical references for the development of safe, efficient, and sustainable active food packaging systems.

**Scheme 1:**
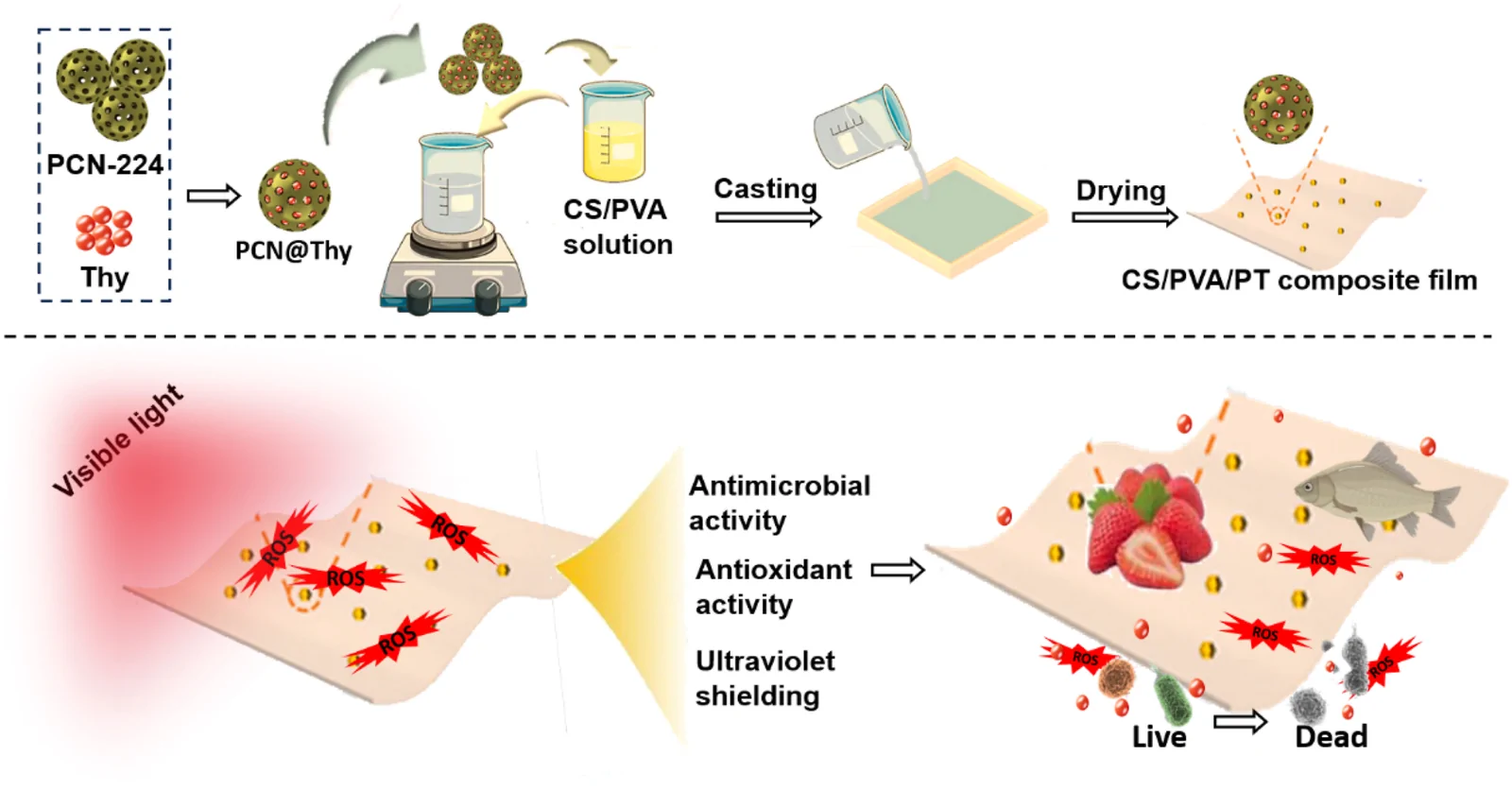
Schematic illustration of preparing CS/PVA/PT composite film and application in food preservation.

## 2 Materials and methods

### 2.1 Reagents and materials

Chitosan (CS, deacetylation degree 80.0%–95.0%, molecular weight 1.61 × 10^5^ g/mol), polyvinyl alcohol (PVA, degree of hydrolysis 98.0-99.0%) and thymol (Thy, biotechnology grade, ≥98.0%) were purchased from Shanghai Macklin Biochemical Technology Co., Ltd. Glutaraldehyde (50 wt%), Zirconyl chloride octahydrate (ZrOCl_2_·8H_2_O, 99 wt%) was brought from Aladdin Biochemical Technology Co., Ltd.; benzoic acid (BA, 99.5 wt%) was provided by Sinopharm Chemical Reagent Co., Ltd.

### 2.2 Preparation and characterization of PCN@Thy nanoparticles

PCN-224 (0.05 g) was synthesized according to previous work (Wang et al., 2023a), followed by dispersing them in an ethanolic solution (≥99.7%, v/v) containing Thy (0.1 g). The mixture was stirred overnight at room temperature. The precipitate was then collected by centrifugation, washed with anhydrous ethanol (≥99.7%, v/v) to remove free Thy. The resulting precipitate was PCN@Thy, and the loading amount of Thy was calculated based on the standard curve.

The ROS generation capability of PCN@Thy under visible light irradiation was evaluated using 2′,7′-dichlorodihydrofluorescein diacetate (DCFH-DA) and singlet oxygen sensor green (SOSG) fluorescent probes (Wang et al., 2023b). Briefly, DCFH-DA (10 mM, Ex: 485 nm) and SOSG (5 μM, Ex: 504 nm) were separately added to the PCN@Thy (120 μg/mL) suspension to detect total ROS and singlet ^1^O_2_, respectively. Phosphate buffered saline (PBS, pH 7.4, 0.01 M) and a pure SOSG solution served as blank controls. The fluorescence intensity (Ex: 485 nm for DCFH-DA; Ex: 504 nm for SOSG) was recorded continuously over a 360 s period under constant visible light irradiation.

### 2.3 Antibacterial activity test of PCN@Thy nanoparticles

*Staphylococcus aureus* (*S. aureus,* AB 91093) and *Pseudomonas aeruginosa* (*P. aeruginosa,* AS1.1129) were chosen as representative Gram-positive and Gram-negative bacteria, respectively, to evaluate the antibacterial activity of the PCN@Thy nanoparticles. This selection was based on the significant pathogenicity and robust biofilm-forming ability of *P. aeruginosa*, making it a critical indicator for assessing the food safety and biofilm eradication capabilities of the packaging materials.

*S. aureus* and *P. aeruginosa* suspensions (10^5^ CFU/mL) were treated with PCN@Thy at varying concentrations (30, 60, 90, and 120 μg/mL). The mixtures were irradiated with visible light (633 nm, 200 W, at a distance of 10 cm from the samples) for 10 min, followed by incubation for 3 h. Subsequently, bacterial survival rates were determined using the plate count method. Bacterial suspensions treated with PBS under dark conditions served as controls.

### 2.4 Fabrication of CS/PVA/PT film

CS (1 g) was dissolved in 40 mL of 1% (v/v) acetic acid solution and stirred at 60 °C until completely dissolved. Separately, PVA (1 g) was dissolved in deionized water under continuous stirring at 80 °C. After cooling to room temperature, the two solutions were mixed at a 1:1 mass ratio to obtain the CS/PVA casting solution (Ali et al., 2024).

To prepare the films, PCN@Thy was added to 10 mL of the CS/PVA casting solution to obtain final mass fractions of 0, 0.3%, 0.6%, 0.9%, and 1.2% (denoted as CS/PVA, CS/PVA/PT 0.3%, CS/PVA/PT 0.6%, CS/PVA/PT 0.9%, and CS/PVA/PT 1.2%, respectively). Higher loadings (>1.2 wt%) were excluded due to severe nanoparticle agglomeration and sedimentation, which prevented the formation of homogeneous films. Glycerol (30%, w/w) was then added as a plasticizer under continuous stirring for 0.5 h. The mixtures were cast into petri dishes, dried at 30 °C for 24 h, and peeled off. The obtained films were then conditioned in a constant temperature and humidity chamber at 25 °C and 50% RH for 48 h prior to analysis.

### 2.5 Characterization of CS/PVA/PT film

The moisture content (MC) of the films was determined according to the previously reported method, with minor modifications (Marand et al., 2021). Film samples (1 cm × 3 cm) were placed in a desiccator containing a saturated Ca (NO_3_)_2_ solution (RH = 55%) for 24 h to reach equilibrium. The obtained samples were then weighed and dried in an oven (110 °C, 24 h). The MC value was calculated based on the weight loss before and after drying.

The films water solubility (WS) was measured following the method described by Marand et al., with slight modifications (Marand et al., 2021). Film samples (1 cm × 3 cm) were dried in an oven (70 °C, 36 h) to determine the initial dry weight (W1). The dried films were then immersed in distilled water and shaken (100 r/min) for 24 h. Afterward, undissolved residue was carefully separated, dried again (110 °C, 24 h), and weighed (W2). Finally, The WS was calculated using the formula:

WS(%)=(W1−W2)/W1×100%.

The tensile strength (TS) and elongation at break (EB) of the films were determined according to a previously reported method (Zhou et al., 2024), with minor modifications, to characterize the mechanical properties of the films. Briefly, film samples were placed in a constant temperature and humidity chamber (25 °C, 50% RH) for 48 h. Subsequently, the films were cut into strips (1 cm × 5 cm), and their thickness was measured using a digital micrometer (accuracy 0.01 mm). Then, the TS and EB were measured by a texture analyzer.

The measurement of water vapor permeability (WVP) and oxygen permeability (OP) was detailed in the supporting information (SI).

### 2.6 The antioxidant activity of CS/PVA/PT film

The DPPH radical scavenging activity of the films was determined according to a previously reported method with minor modifications (Li et al., 2025). The film samples cut into pieces and extracted in 4 mL of distilled water. Subsequently, 2 mL of the film extract was mixed with 2 mL of DPPH solution (0.1 mM) and incubated in the dark for 1.5 h. After that, 200 μL of the reaction mixture was transferred into a 96-well plate to measure the absorbance at 517 nm. An equal volume of PBS replacing the film extract was used as the control to represent 100% free radicals.

To determine the ABTS radical scavenging activity, a previously reported protocol was adopted and modified appropriately (Li et al., 2025). The ABTS working solution was prepared by mixing equal volumes of ABTS solution (7 mM) and potassium persulfate solution (2.45 mM) and incubating in the dark for 16 h. The mixture was then diluted with PBS to an absorbance of 0.7 ± 0.02 at 734 nm. After reacting in the dark for 30 min, the absorbance at 734 nm was measured. An equal volume of PBS was used in place of the film extract as a negative control.

### 2.7 Antimicrobial activity of CS/PVA/PT film

The antibacterial activity of the CS/PVA/PT films against *P. aeruginosa*, *Aeromonas hydrophila* (*A. hydrophila*, 157), and *Vibrio parahaemolyticus* (*V. parahaemolyticus*, 475) was evaluated using the agar diffusion method (Khan et al., 2024). The films were cut into discs (1 cm in diameter), sterilized under UV light (254 nm), and placed onto Luria-Bertani (LB) agar plates inoculated with 50 μL of bacterial suspension (10^8^ CFU/mL). After irradiation with visible light for 10 min, the plates were incubated overnight (37 °C). Finally, the diameters of the inhibition zones were measured using a digital caliper.

The antifungal efficacy of the composite films against *Botrytis cinerea* (*B. cinerea*, B05.10) was evaluated using a mycelial growth inhibition assay (Yuan et al., 2025). The film solution was mixed thoroughly with potato dextrose agar (PDA) medium to prepare the treatment plates. After solidification, a 6 mm mycelial plug of *B. cinerea* was inoculated at the center of each plate. The plates were irradiated with visible light for 10 min and subsequently incubated at 28 °C for 3 days. Colony diameters were recorded every 24 h for 3 days.

### 2.8 Morphological study of bacterial cells

The morphological changes of bacteria were observed by SEM. Briefly, bacterial suspensions were treated with PBS (control), CS/PVA films, or CS/PVA/PT films (1.2 wt% PCN@Thy, under visible light irradiation for 10 min) and incubated for 3 h. Subsequently, the bacterial cells were collected, washed with PBS, fixed with glutaraldehyde (2.5%), and dehydrated using a graded ethanol series (Wang et al., 2024). Finally, the samples were subjected to SEM imaging.

### 2.9 Application of fish preservation

Based on the comprehensive performance evaluation of the CS/PVA/PT films with different PCN@Thy loadings (0.3, 0.6, 0.9, and 1.2 wt%), the formulation containing 1.2 wt% PCN@Thy was selected for the fish preservation experiment due to its optimal antibacterial, antioxidant, and barrier properties. Freshwater-cultured bass were obtained from a local market. The fish were sacrificed and immediately cut into slices (10.5 ± 1.1 g) under aseptic conditions, and then washed with sterile water and exposed to UV irradiation for 30 min. Subsequently, the fish slices were randomly divided into three groups: a control group (unpackaged), a CS/PVA group (wrapped with CS/PVA film), and a CS/PVA/PT group (wrapped with CS/PVA/PT film containing 1.2% PCN@Thy and irradiated with visible light for 10 min prior to storage). All samples were placed in petri dishes and stored at 4 ± 1 °C. Quality indicators were measured on days 0, 3, 6, and 9. Specifically, total volatile basic nitrogen (TVB-N) was determined according to previous method (Chang et al., 2021). Moreover, the pH measurement, total viable count (TVC), firmness, and color difference (Δ*E*) were detailed in the SI.

To assess whether the Zr migration level was within the safe limit, the Zr migration from the CS/PVA/PT film into the fish samples was measured via ICP-MS on days 0 and 9 (Wang et al., 2021).

### 2.10 Application of strawberry preservation

Under aseptic conditions, fresh strawberry fruits (21.5 ± 2.0 g) were washed with sterile water and exposed to UV irradiation for 30 min. Subsequently, they were randomly divided into three groups and placed in packaging cups (7.2 cm in diameter, 7.9 cm in height). The cups were then sealed at the opening with the corresponding films (CS/PVA or CS/PVA/PT) to create a closed headspace environment, while the strawberries remained at the bottom of the cups without direct contact with the films. The control group was left unsealed. For the CS/PVA/PT group, the samples were irradiated with visible light for 10 min prior to storage. All samples were stored at room temperature for 10 days. Sensory appearance, weight, pH, total color difference, and hardness were evaluated on days 0, 2, 4, 6, 8, and 10. Additionally, the measurement of firmness, pH, Δ*E* of strawberries were detailed in SI.

### 2.11 Statistical analysis

All experiment were conducted in triplicate. All obtained data were expressed as the mean value ± standard deviation (SD). Statistical analysis was performed based one-way analysis of variance (ANOVA) by SPSS. The Duncan multi-range test was employed to evaluate the differences between treatment groups (*p* < 0.05).

## 3 Results and discussion

### 3.1 Characterization of PCN@Thy

The morphology of PCN-224 and PCN@Thy was examined by scanning electron microscopy (SEM, SU8100) and transmission electron microscopy (TEM, JEM-2010) (Fig. 1A). Both nanoparticles exhibited a spherical shape. As displayed in Fig. 1B, pure Thy presented an absorption peak at 275 nm (Liu et al., 2024), while PCN-224 showed a typical strong porphyrin absorption peak at 425 nm but lacked a peak at 275 nm (Nie et al., 2020). In contrast, PCN@Thy exhibited not only the characteristic peaks at 425 nm and 525 nm, but also a distinct peak at 275 nm. This indicated that Thy was successfully loaded into PCN-224, and the loading efficiency was 5.88%. Dynamic light scattering **(**DLS) analysis was performed on a Malvern Zeta sizer (ZS90) (Fig. 1C) revealed that the size of PCN@Thy was mainly distributed between 142 and 220 nm, which was larger than that of PCN-224 (122-190 nm) due to the loading of Thy. Additionally, the polydispersity index (PDI) of PCN@Thy was 0.245. Furthermore, a comparison of the XRD spectra (Fig. 1D) showed that PCN@Thy retained all the characteristic diffraction peaks of PCN-224 without any residual peaks from Thy. This confirmed that the crystalline structure of PCN-224 was well preserved after the loading process. The PDI value of 0.245 (<0.3) indicates a monodisperse system with minimal aggregation, ensuring the homogeneity of the film-forming suspension and the uniformity of the final films. The FTIR spectra (Fig. 1E) showed that both PCN-224 and PCN@Thy exhibited characteristic vibration peaks attributed to C=O and C=C groups at 1712 cm^−1^ and 1658 cm^−1^, respectively. Notably, the characteristic peaks of Thy were barely visible in the PCN@Thy spectrum, indicating that Thy had been successfully loaded into the pores of PCN-224 (Min et al., 2021). Moreover, the characteristic peaks of MOF in the CS/PVA/PT film were not obvious, implying that the nanoparticles were well-distributed within the film matrix. Moreover, TEM element mapping manifested that Zr, C, N, and O elements were uniformly distributed within the same nanoparticle. The distribution of Zr and N corresponds to the framework structure of PCN-224, while the uniform distribution of O elements indicated that the introduction of Thy did not disrupt the material's homogeneity, with no obvious phase separation observed (Fig. 1F). This further confirmed the successful preparation of the PCN@Thy composite.

**Fig. 1:**
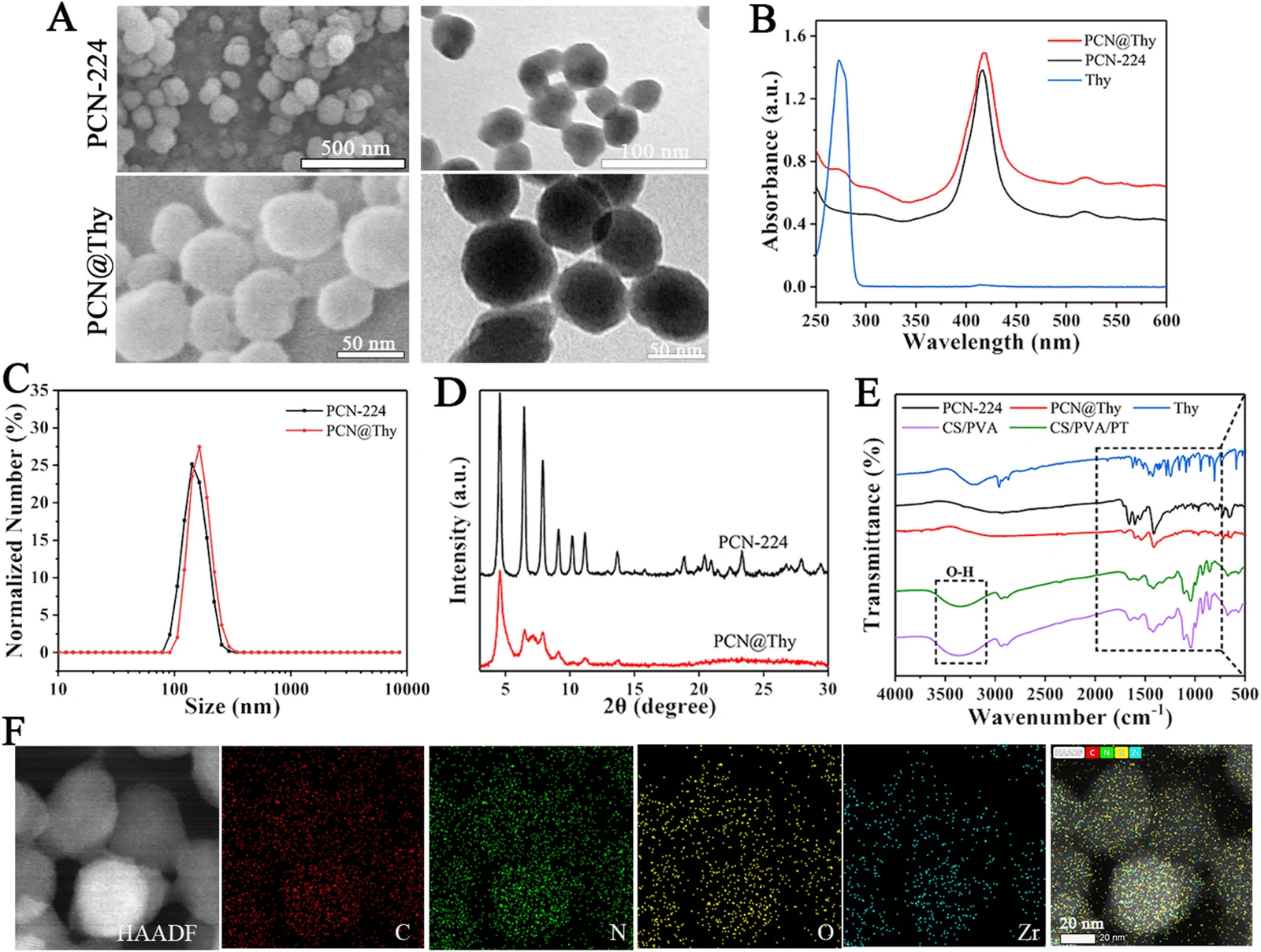
(A) TEM and SEM images of PCN@Thy. (B) UV−vis absorption spectra, (C) hydrodynamic size, and (D) XRD of PCN-224 and PCN@Thy. (E) FT-IR spectra of PCN-224, Thy, CS/PVA, PCN@Thy, and CS/PVA/PT. (H) Element mapping of PCN@Thy. Scale bar, 20 nm.

Furthermore, the ROS generation capability of PCN@Thy was determined employing DCFH-DA. The fluorescence intensity of PCN@Thy markedly increased under visible light irradiation (Fig. 2A), indicating that light could effectively promote the generation of ROS. To specifically identify the type of ROS, the ^1^O_2_-specific probe SOSG was used. The results showed that negligible fluorescence was observed in the PBS control group and SOSG-only groups, whereas the PCN@Thy group exhibited a robust fluorescence enhancement. This strongly confirmed the efficient generation of ^1^O_2_ by PCN@Thy under visible light excitation (Fig. 2B).

**Fig. 2:**
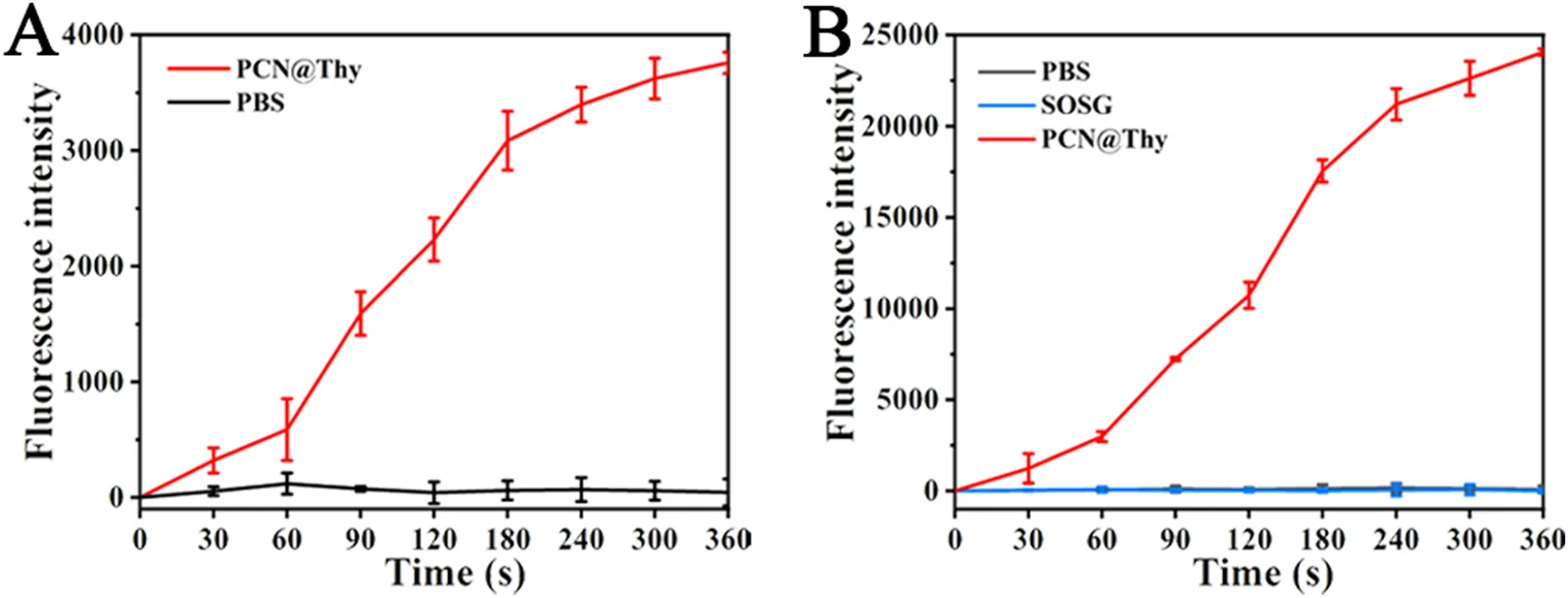
(A) ROS generation of PCN@Thy under light irradiation using DCFH-DA as the sensor. (B) ^1^O_2_ generation of PCN@Thy under light irradiation.

### 3.2 Assessment of antibacterial activity of PCN@Thy nanoparticles

The antibacterial activity of PCN@Thy was evaluated utilizing the plate counting method. As illustrated in Fig. 3A, both *S. aureus* and *P. aeruginosa* formed numerous colonies in the control group, which was defined as 100% survival. As shown in Fig. 3B, treatment with free Thy alone resulted in limited inhibitory effects, with survival rates decreasing only moderately from 89.02% to 46.32% for *S. aureus* and from 86.87% to 53.03% for *P. aeruginosa* as the concentration increased from 30 to 120 μg/mL. Similarly, PCN-224 alone showed modest concentration-dependent activity, reducing *S. aureus* survival from 51.07% to 20.45% and *P. aeruginosa* survival from 69.43% to 13.60% over the same concentration range. In striking contrast, PCN@Thy exhibited a much more pronounced concentration-dependent antibacterial effect. Under identical conditions, the survival rate of *S. aureus* dropped sharply from 47.86% to 9.17%, while that of *P. aeruginosa* decreased from 59.12% to 9.88%. At the highest concentration tested (120 μg/mL), the survival rate of *S. aureus* in the PCN@Thy group was only 22% and 0.19% of that observed in the Thy and PCN-224 groups, respectively. Similarly, for *P. aeruginosa,* the survival rate was reduced to 45% and 0.73% of the corresponding controls. These results clearly demonstrate the superior antibacterial efficacy of the PCN@Thy composite compared to its individual components. The dramatic reduction in survival can be attributed to a dual mechanism: PCN-224 generates ROS under light, causing bacterial apoptosis by damaging cell membranes, proteins, and DNA (Deng et al., 2025); simultaneously, the sustained release of the natural antibacterial agent Thy from the PCN-224 carrier further amplifies the bactericidal effect. Previous studies have demonstrated that Thy exerts potent antibacterial activity against both *S. aureus* and *P. aeruginosa* by disrupting cell membrane integrity, increasing membrane permeability, and inducing oxidative stress (Li et al., 2022; Nazzaro et al., 2013).

**Fig. 3:**
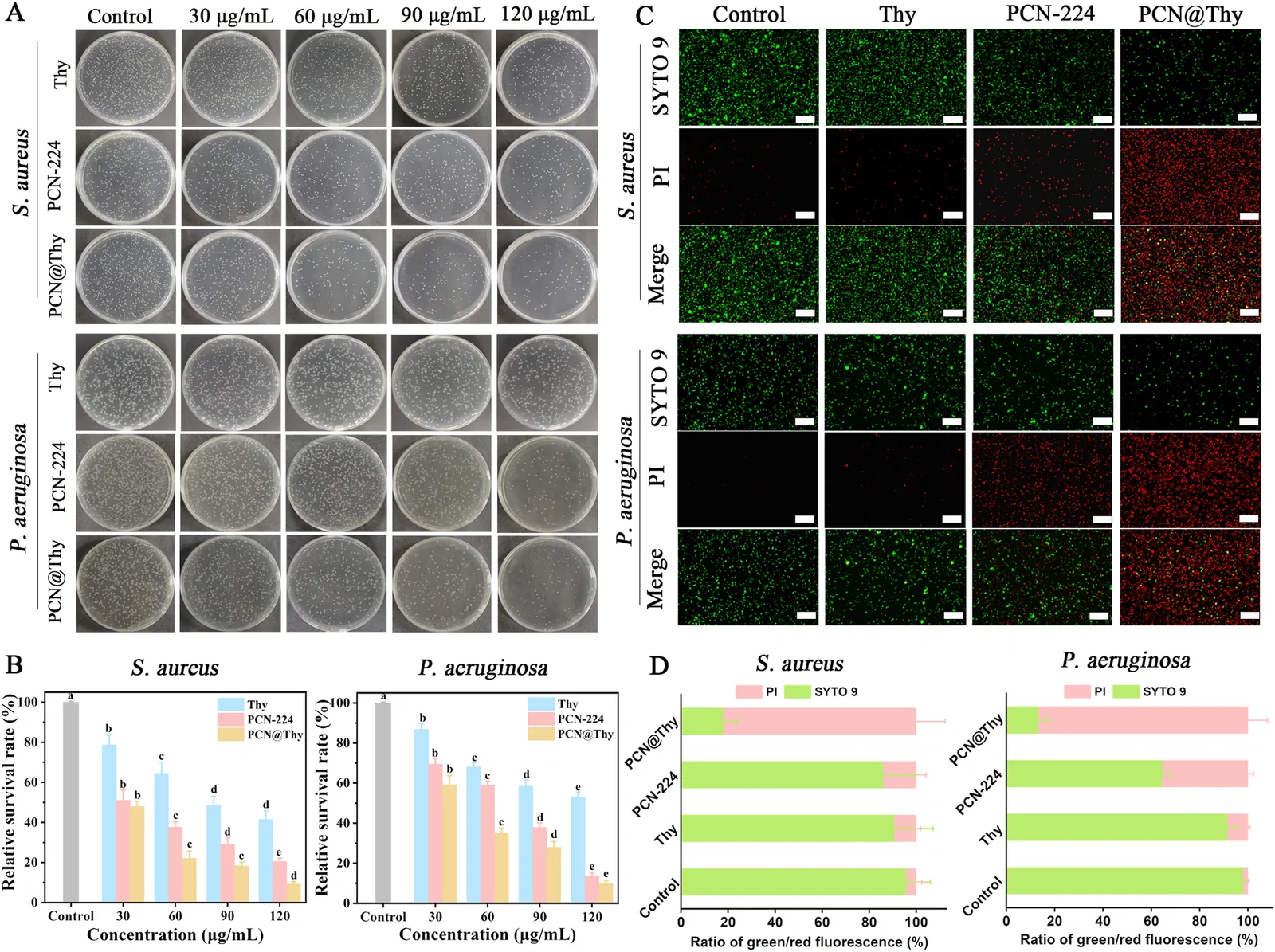
Photographs of the agar plates (A) and corresponding survival rate (B) of *S. aureus* and *P. aeruginosa* treated with different materials. Representative live/dead images (C) and and (D) the ratio of green/red fluorescence of *S. aureus* and *P. aeruginosa* treated with different materials. Scale bar, 100 μm. (Data are means ± SD (n = 3). Different letters in each column show significant difference at the 5% level in Duncan's test (*p* < 0.05).

Moreover, the antibacterial activity of PCN@Thy was assessed using a live/dead staining assay. SYTO 9 (a green-fluorescent nucleic-acid stain) and propidium iodide (PI, a red-fluorescent nucleic-acid stain) were employed to stain live (green fluorescence) and dead bacteria (red fluorescence), respectively. As manifested in Fig. 3C and D, the control group exhibited predominantly green fluorescence, indicating intact cell membranes and active bacteria. Similar results were observed for the Thy and PCN-224 groups; although red fluorescence increased slightly, green fluorescence still dominated (90.70% and 85.98%, respectively), suggesting that only a fraction of the bacteria were inactivated. In contrast, the PCN@Thy group presented a completely opposite phenomenon with red fluorescence accounting for up to 81.95%. This strongly confirmed that PCN@Thy treatment with visible light irradiation caused massive bacterial inactivation, which aligns well with the quantitative plate-counting results above.

### 3.3 Evaluation of anti-biofilm activity of PCN@Thy nanoparticles

The formation of biofilms plays a critical role in the development of bacterial resistance (Elafify et al., 2024). An ideal antibacterial agent should not only effectively kill planktonic (free-floating) bacteria but also inhibit biofilm formation or eradicate mature biofilms. As expressed in Fig. 4A–*S aureus* biofilms completely covered the bottom of the wells in the control group. In contrast, PCN@Thy treatment significantly inhibited biofilm formation in a concentration-dependent manner. For example, at a concentration of 120 μg/mL, the biofilm biomass was significantly (*p* < 0.05) reduced to 60% of that of the control group (Fig. 4B). A similar trend was observed for *P. aeruginosa*. Since mature biofilms continuously release pathogenic bacteria, their effective removal is of vital importance(Rossi et al., 2022). The study found that PCN@Thy also exhibited excellent biofilm clearance efficacy in a concentration-dependent manner (Fig. 4A). Compared to the control group (with an OD_590_ value of 3.1), treatment with 120 μg/mL PT significantly decreased the remaining biofilm mass to 2.1 (Fig. 4C).

**Fig. 4:**
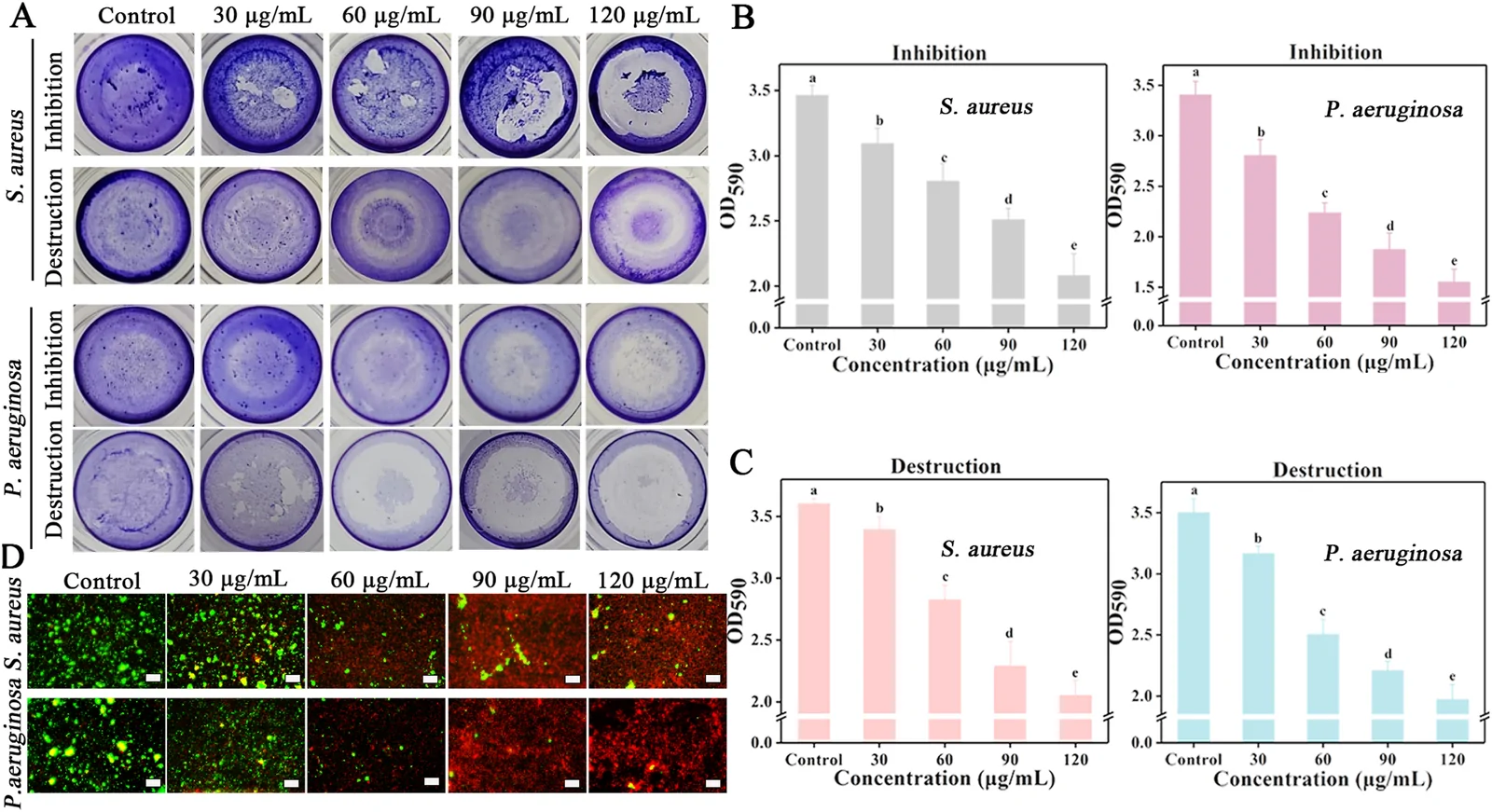
(A) Pictures of crystal-violet-stained biofilm and corresponding amount of (B) biofilm formation and (C) biofilm destruction of *S. aureus* and *P. aeruginosa* treated with different films. (D) Representative live/dead images of forming biofilms of *S. aureus* and *P. aeruginosa*, scale bar, 100 μm. (Data are means ± SD (n = 3). Different letters in each column show significant difference at the 5% level in Duncan's test (*p* < 0.05).

Furthermore, the anti-biofilm capability of PT was confirmed via confocal laser scanning microscopy (CLSM) employing fluorescein isothiocyanate-concanavalin A (FITC-ConA) and PI as fluorescent dyes. As exhibited in Fig. 4D, the control group showed strong green fluorescence, indicating the presence of abundant extracellular polysaccharides (EPS). After treatment with PCN@Thy, both green and red fluorescence signals were detected. Notably, as the PCN@Thy concentration elevated, the green fluorescence gradually diminished, while the red fluorescence (representing dead bacteria) progressively intensified. Since EPS tightly encapsulates bacteria to maintain the three-dimensional structure of the biofilm (Wang et al., 2023a), we speculate that PCN@Thy initially disrupts the EPS matrix. This degradation weakens the physical barrier of the biofilm, thereby facilitating the penetration of antibacterial agents and effectively eradicating bacteria within the deeper layers.

### 3.4 Fabrication and characterization of CS/PVA/PT films

#### 3.4.1 The morphology of CS/PA nanocomposite films

The surface and cross-sectional microstructures of the CS/PVA/PT films were examined via SEM. As displayed in Fig. 5A, the surface of CS/PVA film was smooth and uniform, with no obvious protrusions, voids or pores, implying good compatibility of the CS and PVA molecules. The CS/PVA/PT 0.3% film presented a similar smooth morphology. However, as the PCN@Thy content increased further, the surface morphology underwent significant changes, presenting a rougher structure accompanied by aggregated particles. This agglomeration became more pronounced at higher nanoparticle loadings. While the SEM images provide a qualitative assessment of this increased roughness, future studies will employ atomic force microscopy (AFM) to obtain precise quantitative roughness parameters for the films, especially the CS/PVA/PT 1.2% film, to further validate these morphological changes. This effect may be attributed to the uneven distribution of PCN@Thy nanoparticles within the polymer matrix, which aligns with previous findings (H. Wang et al., 2025a). Despite this surface roughness, all samples maintained a fundamentally dense and continuous internal structure without visible cracks or large voids. A similar morphological trend was observed in the cross-sectional images.

**Fig. 5:**
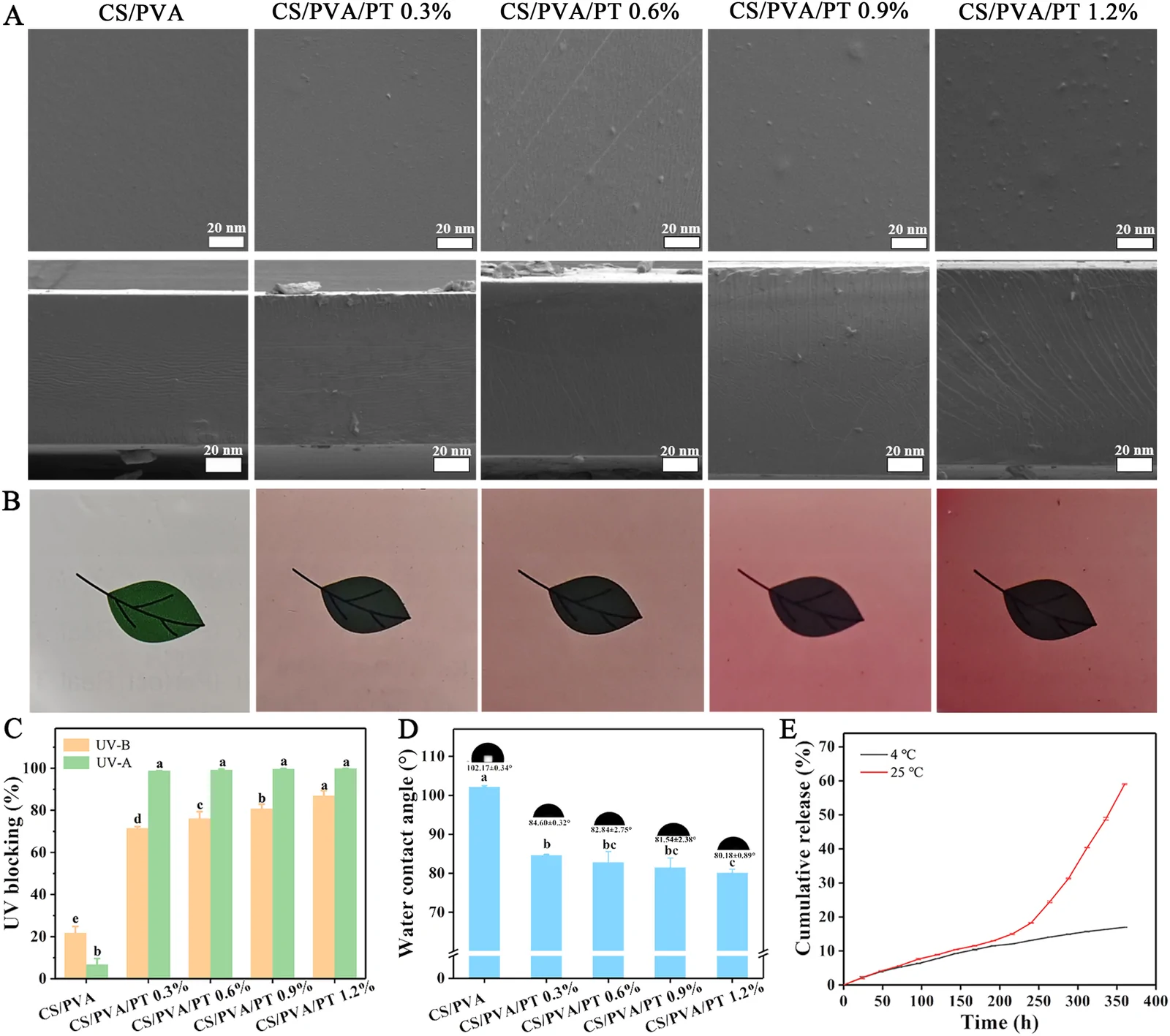
(A) SEM micrographs of surfaces and cross-sections, (B) Appearance photographs, (C) UV-blocking efficiency, and (D) images of water contact angle of CS/PVA-based films incorporated with different PCN@Thy. (E) Thy release behaviors. (Data are means ± SD (n = 3). Different letters in each column show significant difference at the 5% level in Duncan's test (*p* < 0.05).

#### 3.4.2 Optical performance and color

The transparency and color of packaging films are crucial for practical applications, as they significantly influence consumer acceptance (Tan et al., 2024). As depicted in Fig. 5B, all films exhibited smooth and uniform surfaces. Notably, compared to the colorless pure CS/PVA film, the composite films presented a distinct red color that deepened with increasing PCN@Thy content, which is primarily attributed to the purplish-red characteristic of PCN-224 (Burger et al., 2022). As listed in Table 1, the pure CS/PVA film exhibited the highest L* value. With increasing PCN@Thy loading, the L* value gradually decreased, indicating reduced brightness, while the a* and b* values simultaneously elevated due to the inherent color of the nanoparticles. Color difference analysis revealed that the ΔE value increased progressively, confirming that the introduction of PCN@Thy significantly altered the optical properties of the nanocomposites. The wavelength of 660 nm was selected to evaluate the film transparency in the visible region, which is closely related to consumer visual perception of packaged products. Additionally, the pure CS/PVA film exhibited excellent transparency, with a light transmittance at 660 nm (T_660_) as high as 97.46%. However, the incorporation of PCN@Thy significantly (*p* < 0.05) reduced the transparency of the films, with the T_66**0**_ of the highest concentration group dropping to 65.46%.

**Table 1:** Optical characteristics of CS/PVA/PT films.

Film	L*	a*	b*	ΔE	T_280_ (%)	T_660_ (%)
CS/PVA	93.86 ± 0.06^a^	−0.38 ± 0.09^e^	0.46 ± 0.31^c^	0.71 ± 0.06^e^	85.19 ± 0.61^a^	97.46 ± 1.34^a^
CS/PVA/PT 0.3%	86.01 ± 0.29^b^	0.64 ± 0.33^d^	1.22 ± 0.18^bc^	8.34 ± 0.30^d^	56.14 ± 3.71^b^	80.46 ± 1.10^b^
CS/PVA/PT 0.6%	82.07 ± 0.45^c^	3.75 ± 0.28^c^	2.00 ± 1.13^b^	12.88 ± 0.52^c^	27.75 ± 1.37^c^	77.98 ± 0.32^bc^
CS/PVA/PT 0.9%	76.72 ± 0.30^d^	11.08 ± 0.36^b^	3.18 ± 0.35^a^	20.97 ± 0.46^b^	21.40 ± 0.78^d^	75.69 ± 0.35^c^
CS/PVA/PT 1.2%	70.75 ± 0.42^e^	14.95 ± 0.36^a^	3.49 ± 0.25^a^	28.07 ± 0.56^a^	18.00 ± 1.00^d^	65.46 ± 2.70^d^

Data are means ± SD (n = 3). Different letters in each column show significant difference at the 5% level in Duncan's test (*p* < 0.05).

On the other hand, the wavelength of 280 nm was chosen to assess the UV-shielding performance of the films, as this region is particularly critical for protecting light-sensitive food components from photo-oxidation. The UV-shielding capacity of the films was determined by the light transmittance at 280 nm (T_280_). Due to the lack of UV-absorbing functional groups in the CS molecular structure (Zhang et al., 2019), the pristine CS/PVA film exhibited poor light-blocking performance (T_280_ = 85.19%). However, the introduction of PCN@Thy significantly enhanced the UV-shielding properties. Specifically, T_280_ dropped sharply to 18.00%, confirming a strong shielding capability in the UV-C region. Notably, with the addition of 1.2% PCN@Thy, the composite film blocked 99.89% of UV-A (320-400 nm) and 86.94% of UV-B (280-320 nm) radiation (Fig. 5C) (Khan et al., 2023). This remarkable improvement is primarily attributed to the strong UV absorption characteristics of the PCN-224 framework. In addition, the light scattering effect caused by the dispersed nanoparticles within the film matrix further contributes to the reduced UV transmittance (Chen et al., 2025).

Ultraviolet and visible light radiation can accelerate food oxidation deterioration and quality degradation by triggering free-radical chain reactions that damage unsaturated fatty acids and vitamins (Guo et al., 2026). Specifically, UV-A and UV-B irradiation trigger free-radical chain reactions in food matrices. For aquatic products such as fish fillets, UV initiates lipid peroxidation, generating peroxyl radicals that decompose unsaturated fatty acids, leading to rancidity, discoloration, and texture degradation. For anthocyanin-rich fruits like strawberries, UV exposure directly induces photodegradation of anthocyanins and accelerates color fading. Furthermore, it promotes the accumulation of ROS, aggravating cell membrane oxidative damage and accelerating fruit senescence and rotting. In this study, the pristine CS/PVA film exhibited poor UV-blocking capacity (T_280_ = 85.19%), whereas the incorporation of 1.2 wt% PCN@Thy dramatically reduced T_280_ to 18.00%, achieving 99.89% UV-A blocking and 86.94% UV-B blocking. This remarkable performance originates from the strong UV-absorbing porphyrin chromophores of PCN-224, which synergize with the light-scattering effect of dispersed nanoparticles to collectively establish an efficient UV barrier. To objectively contextualize the advantages of this UV-shielding performance, we compared it with representative biopolymer-based UV-blocking packaging materials reported in recent literature. For instance, TiO_2_/chitosan composite films have been reported to achieve approximately 92% UV-A blocking at a TiO_2_ loading of 2.0 wt% (Zhang et al., 2019), while N, P-doped carbon dots/starch films have demonstrated ∼90% UV-A shielding capacity (Khan et al., 2023). In contrast, CS/PVA/PT 1.2% film achieves superior UV-A shielding efficiency (99.89%) at a substantially lower filler content (1.2 wt%). Moreover, unlike many inorganic nanoparticle-based systems that severely compromise film transparency, our film maintains a visible-light transmittance (T_660_) of 65.46%, striking a favorable balance between protective functionality and consumer visual appeal. Collectively, these results demonstrate that the incorporation of PCN@Thy optimizes the optical properties of the composite film, endowing it with excellent light-shielding capability. This function, together with its sustained antibacterial and antioxidant activities, constitutes a multi-tiered food protection barrier that intervenes at the initiation stage of photo-oxidative deterioration, thereby effectively extending the shelf life of food products.

#### 3.4.3 Water contact angle (WCA)

WCA is an important indicator for evaluating the hydrophilic/hydrophobic properties of film surfaces. As illustrated in Fig. 5D, the pure CS/PVA film exhibited the largest water contact angle (102.17 ± 0.34°), indicating a hydrophobic surface 19(WCA > 90°) (Polinarski et al., 2024). With the incorporation of PCN@Thy, the WCA gradually decreased, dropping to 80.18 ± 0.89° at an addition of 1.2%, which demonstrates a significant shift towards hydrophilicity. Generally, the surface wettability of composite films is influenced by the loaded nanofillers and the polymer matrix, specifically through surface roughness and functional groups (Khan et al., 2023). In this case, the increased surface roughness induced by PCN@Thy (Fig. 5A) played a role. More importantly, the hydrophilic hydroxyl groups inherently present in both PCN-224 and thymol fundamentally enhanced the surface hydrophilicity (RR, 2019). This aligns with literature reports stating that adding hydrophilic additives typically reduces the WCA of biopolymer-based packaging films (H. Wang et al., 2025a), thereby increasing surface wettability.

#### 3.4.4 The release of thy

The release of Thy from the CS/PVA/PT film is closely related to its preservation performance. As displayed in Fig. 5E, Thy release was significantly influenced by temperature. Within the initial 200 h, the release rates at both 4 °C and 25 °C were relatively slow. However, after 200 h, the release rate at 25 °C significantly increased, significantly outpacing the 4 °C group and continuing until 350 h. This demonstrates an excellent long-term sustained-release capability. Importantly, this prolonged release profile is highly advantageous for food preservation, as it ensures a continuous supply of antibacterial agents during storage.

#### 3.4.5 Moisture content (MC) and water solubility (WS)

To assess the water resistance of the films, their MC and WS were measured. It can be seen that the incorporation of PCN@Thy significantly affected the MC of the CS/PVA film (Fig. 6B). Notably, at higher PCN@Thy loadings, the MC of the composite film (23.42%) was significantly (*p* < 0.05) lower than that of the control group (33.22%). This is likely because the uniformly dispersed nanoparticles occupy the space originally available for water adsorption, thereby reducing the film's capacity to capture and retain water molecules (Tirgarian et al., 2023). Moreover, WS is a key factor determining the structural integrity of films during food preservation, especially for high-humidity applications (Ismail et al., 2019). As expressed in Fig. 6C, the CS/PVA film exhibited the highest WS (39.24%). The addition of PCN@Thy effectively declined the WS, with the 1.2% PCN@Thy composite film showing a reduction to 21.05%. This was primarily attributed to the interaction between PCN@Thy and the CS matrix, which reduced the water binding sites and increased the crosslinking density, thereby effectively inhibiting film dissolution (Marand et al., 2021). These results indicate that the optimized CS/PVA/PT formulation with 1.2 wt% PCN@Thy possesses excellent water-resistant properties, making it a promising candidate for food packaging applications.

**Fig. 6:**
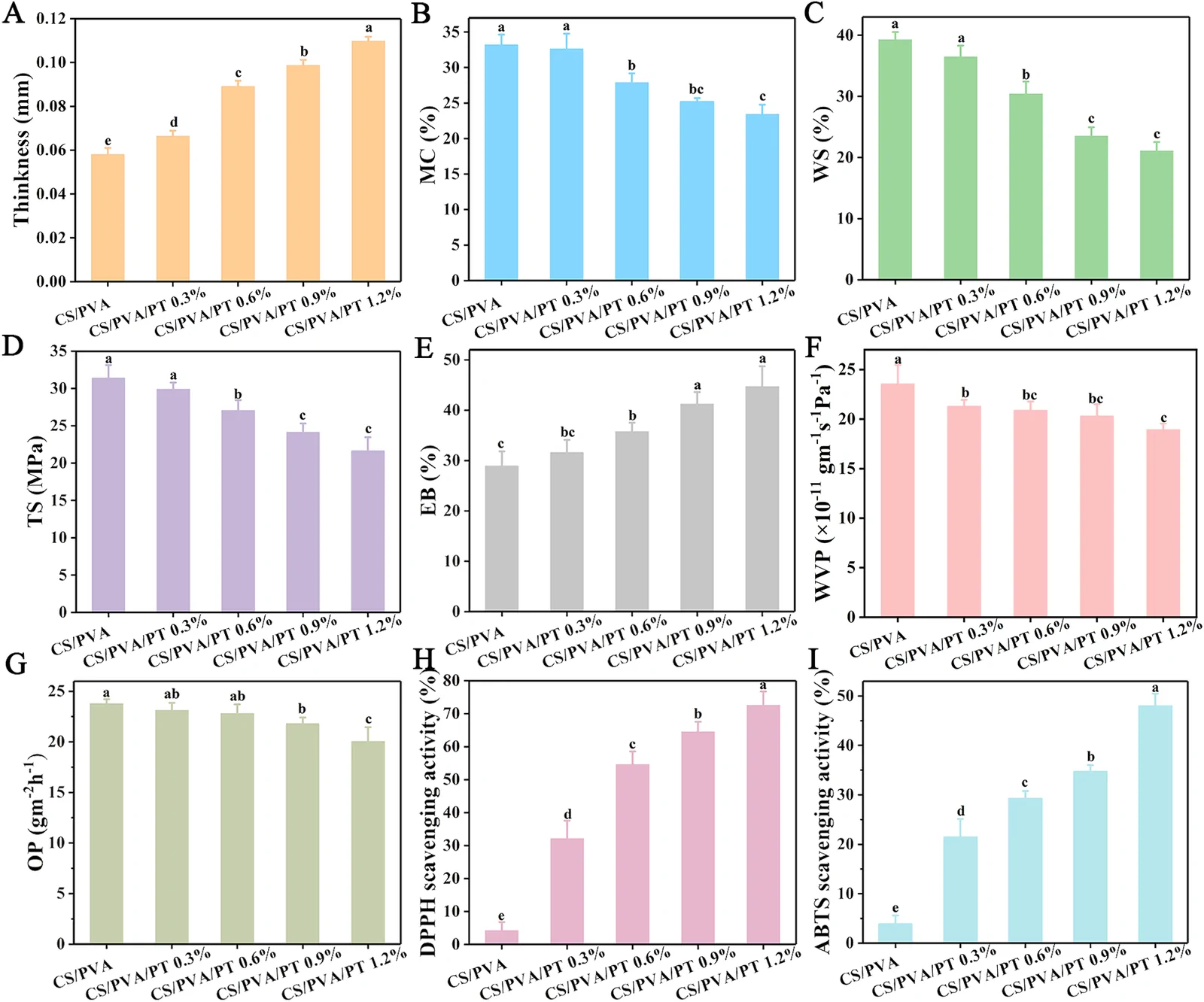
(A) Thickness, (B) MC, (C) WS, (D) TS, (E) EB, and (F) WVP, (G) OP, (H) DPPH, and (I) ABTS free radical scavenging capability of different films. (Data are means ± SD (n = 3). Different letters in each column show significant difference at the 5% level in Duncan's test (*p* < 0.05).

#### 3.4.6 Mechanical properties

The mechanical characteristics of the films, including thickness, EB, TS, are depicted in Fig. 6A. The thickness of the pure CS/PVA film significantly (*p* < 0.05) increased from 0.058 mm to 0.110 mm with the incorporation of PCN@Thy. This can be attributed to the presence and aggregation of PCN@Thy nanoparticles within the polymer matrix (Shah et al., 2019). Packaging materials must exhibit suitable mechanical strength and extensibility to resist external stress and maintain integrity during transportation, storage, and processing, which is of vital importance for food protection (Feng et al., 2024). As core indicators of film performance, TS reflects the maximum stress a film can withstand, while EB represents its flexibility and stretchability before rupture (Ma et al., 2024). Together, they determine the applicability of the films and are of great significance in food packaging applications. As illustrated in Fig. 6D and E, the pure CS/PVA film exhibited high tensile strength but relatively low extensibility (TS = 31.38 MPa; EB = 28.91%), which is attributed to the hydrogen bond network formed between CS and PVA molecules. The incorporation of PCN@Thy significantly altered the mechanical properties of the CS/PVA film. Specifically, the addition of PCN@Thy significantly altered these mechanical properties; specifically, it significantly (*p* < 0.05) reduced the TS while simultaneously increasing the EB. The reduction in TS is likely because the nanoparticles weakened the hydrogen bond interactions between CS/PVA chains, and particle aggregation disrupted the continuity of the matrix, leading to local stress concentration.

Cross-sectional SEM observations (Fig. 5A) reveal pronounced nanoparticle aggregation within the polymer matrix at higher PCN@Thy loadings. These aggregated domains act as internal structural defects and generate local stress-concentration centers under tensile force, which are responsible for the deterioration of tensile strength (Le et al., 2025). FTIR spectra further provide molecular-level evidence for the change in hydrogen-bond interactions. The broad O–H stretching band of pure CS/PVA, located at 3200–3600 cm^−1^, shifts toward higher wavenumbers after PCN@Thy incorporation, demonstrating partial disruption of the original intermolecular hydrogen-bond network (Hajji et al., 2016; Lusiana et al., 2025). The breakdown of this compact network increases the freedom of polymer segmental motion, which contributes to the elevated elongation at break (Coleman et al., 2006). Collectively, these results indicate that the CS/PVA/PT film possesses favorable overall mechanical properties for practical packaging applications.

#### 3.4.7 Water vapor permeability (WVP) and oxygen permeability (OP)

WVP and OP are key indicators of barrier properties in food packaging films (Xin et al., 2020). As exhibited in Fig. 6F, the pure CS/PVA film exhibited the highest WVP of 23.55 × 10^−11^ g m^−1^s^−1^Pa^−1^, indicating the poorest water vapor barrier performance. The addition of PCN@Thy improved the water vapor barrier performance of the film in a concentration-dependent manner, declining the WVP value to 18.94 × 10^−11^ g m^−1^s^−1^Pa^−1^. The OP of the pure CS/PVA film was 23.76 gm^−2^h^−1^, whereas the CS/PVA/PT 0.3% film displayed a value of 23.10 gm^−2^h^−1^. As the PCN@Thy content increased, the OP consistently decreased, ultimately dropping to 20.04 gm^−2^h^−1^ for the CS/PVA/PT 1.2% film (Fig. 6G), demonstrating that an appropriate amount of PCN@Thy improves the oxygen barrier property. This enhancement is mainly attributed to the enhanced interaction between the nanoparticles and the polymer matrix, which create a denser network structure. This dense matrix forces oxygen molecules to navigate a tortuous path (Xu et al., 2019), thereby reducing the oxygen permeability rate. Among all tested formulations, the CS/PVA/PT 1.2% film exhibited the lowest OP and WVP, confirming its excellent potential for preventing food oxidation in practical packaging applications.

#### 3.4.8 Antioxidant analysis

Packaging films with excellent antioxidant properties can mitigate oxidative spoilage in food. As revealed in Fig. 6H and I, the CS/PVA film exhibited limited antioxidant activity, demonstrating a relatively weak capacity to scavenge DPPH and ABTS free radicals. In contrast, the antioxidant capacity of the CS/PVA/PT films was significantly (*p* < 0.05) enhanced, showing a clear concentration-dependent upward trend. For example, the DPPH and ABTS scavenging rates of the CS/PVA/PT 1.2% film reached 72.54% and 47.92%, respectively. This substantial enhancement is fundamentally driven by the potent antioxidant nature of thymol (Thy) loaded within the composite (Chen et al., 2025). Furthermore, when combined with the sustained-release profile of Thy from the film matrix, this synergistic effect guarantees long-term and robust oxidation resistance for practical food packaging applications.

### 3.5 Antimicrobial properties of the composite films

Considering the target application of this film for chilled-fish preservation, three typical aquatic-relevant spoilage and pathogenic bacterial strains (*P. aeruginosa*, *A. hydrophila*, *V. parahaemolyticus*) together with *B*. *cinerea* (a major fungus causing strawberry postharvest decay) were selected as representative test strains to evaluate the antimicrobial efficacy of the CS/PVA/PT film. As illustrated in Fig. 7A and B, inhibition zones were observed around both the CS/PVA and CS/PVA/PT films for all three tested bacteria. The CS/PVA film exhibited the smallest inhibition zones, which can be attributed solely to the inherent antibacterial properties of CS (Flórez et al., 2022). In contrast, the inhibition zone diameter of the CS/PVA/PT films significantly increased (*p* < 0.05) in a concentration-dependent manner. For instance, against *P. aeruginosa*, the inhibition zone diameter of the CS/PVA film was only 0.5 mm, whereas those for the CS/PVA/PT 0.3% and 1.2% films significantly expanded to 1.12 mm and 1.36 mm, respectively. This remarkable antibacterial efficacy is attributed to a multi-target mechanism involving the intrinsic activity of CS, the sustained release of thy, and the oxidative stress induced by ROS generation from photoactivated PCN-224 (Wang et al., 2023a).

**Fig. 7:**
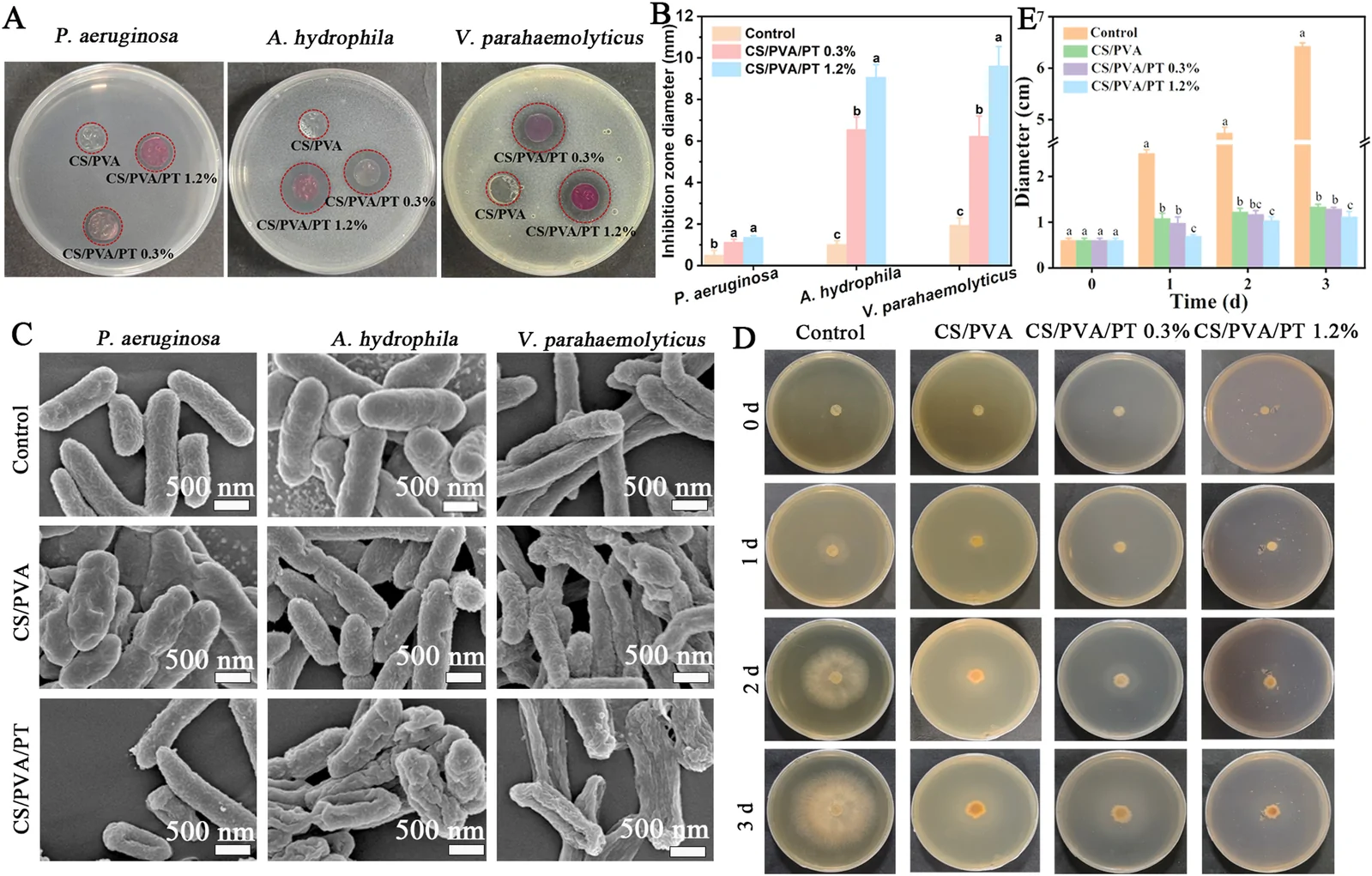
(A) Radius of the inhibition zone, (B) the corresponding inhibition zone data, and (C) typical SEM images of *P. aeruginosa*, *A. hydrophila*, and *V. parahaemolyticus* treated with different films. (D) Colony pictures of *B. cinerea* treated with different films and (E) corresponding colony diameter. (Data are means ± SD (n = 3). Different letters in each column show significant difference at the 5% level in Duncan's test (*p* < 0.05).

Moreover, SEM analysis confirmed that the CS/PVA/PT film induced severe morphological damage to bacterial cells, exhibiting obvious shrinkage, collapse, and rupture (Fig. 7C). Since the intact cell membrane is a critical barrier essential for maintaining cellular homeostasis and energy transduction (Wang et al., 2024), this loss of membrane integrity severely compromises permeability. This triggers an osmotic imbalance and the leakage of intracellular constituents, ultimately leading to cell lysis and death (Wang et al., 2023b). Hence, these combined results demonstrate that the CS/PVA/PT film effectively eradicates bacteria primarily through the robust disruption of membrane integrity, which is driven by the release of Thy and visible-light-induced ROS generation.

To provide quantitative insight into the bactericidal mechanism observed in SEM, supplementary analyses of intracellular ROS, protein leakage, and membrane potential were conducted. As shown in Fig. S1, the CS/PVA/PT group exhibited strong green fluorescence under visible light, indicating substantial ROS generation and subsequent oxidative damage. Concurrently, the film caused significantly higher protein leakage compared to the control (Fig. S2), confirming severe disruption of membrane permeability. Furthermore, membrane potential monitoring revealed a significant decrease in fluorescence intensity upon treatment with PCN@Thy (Fig. S3), indicative of membrane depolarization and dysfunction. Collectively, these quantitative results, combined with the SEM morphological evidence, comprehensively illustrate the dual bactericidal pathway involving ROS-induced oxidative damage and Thy-triggered membrane disruption.

As shown in Fig. 7D, the colony diameter of *B. cinerea* in all groups expanded progressively over time. Compared with the control group, the expansion was significantly suppressed (*p* < 0.05) in both the CS/PVA and CS/PVA/PT treatment groups, with the most pronounced inhibition observed in the CS/PVA/PT 1.2% group. For instance, by day 3, the colony diameter in the control group had reached 6.42 cm, whereas the diameters in the CS/PVA, CS/PVA/PT 0.3%, and CS/PVA/PT 1.2% groups were 1.33 cm, 1.29 cm, and 1.11 cm, respectively. These values accounted for 20.76%, 20.07%, and 17.30% of the control diameter, corresponding to antifungal inhibition rates of 79.24%, 79.93%, and 82.70%, respectively (Fig. 7E). These results clearly suggest that the CS/PVA/PT film possesses potent antifungal activity. In summary, these findings demonstrate that the CS/PVA/PT film exhibits broad-spectrum antimicrobial activity against both bacteria and fungi, highlighting its immense potential as an active food packaging material.

### 3.6 Biosafety evaluation of composite film

#### 3.6.1 Biocompatibility

Safety is a fundamental characteristic for food packaging materials. Therefore, the biocompatibility of the composite films was determined by hemolysis and MTT assays (Materials, 2000; Standardization, 2009). The results demonstrated that erythrocytes in the CS/PVA film extract remained largely intact with negligible morphological damage, and the hemolysis rate was merely 0.59%. Similarly, the CS/PVA/PT film extracts exhibited low hemolysis rates; even for the CS/PVA/PT 1.2% group, the value was only 1.74% (Fig. 8A), well below the 5% safety threshold (Sun et al., 2020), indicative of reliable blood compatibility. Moreover, cell viability remained above 98% upon exposure to the various film extracts (Fig. 8B), confirming that the films are safe and non-toxic.

**Fig. 8:**
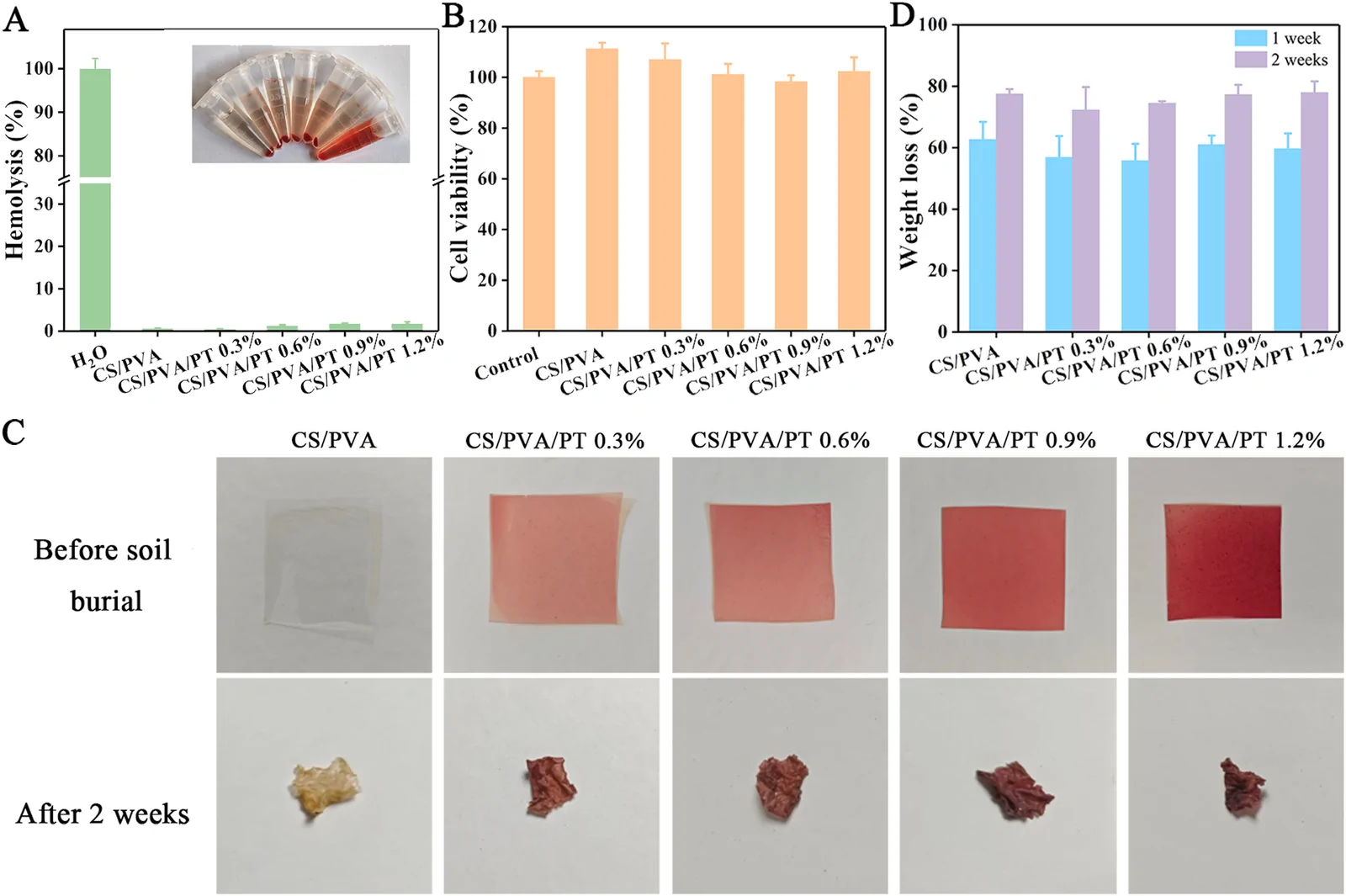
(A) Cell cytotoxicity of CCK8 cells treated with extract solutions from different films. (B) Hemolysis analysis of extract solutions from different films. (C) The weight loss rate of the film and images (D) of the films on different days.

#### 3.6.2 Biodegradability

To evaluate the biodegradation performance of the composite films in a natural environment, their decomposition in soil was investigated. The results indicated that all films exhibited rapid and significant weight loss within the first week (Fig. 8C). This was primarily attributed to the disruption of the polymer network by soil microorganisms and the rapid penetration of water molecules, which facilitated polymer chain scission and led to noticeable physical deterioration (Indumathi and Rajarajeswari, 2019). Specifically, over the burial period, the weight loss of the pure CS/PVA film ranged from 62.67% to 77.49%, while the CS/PVA/PT composite films showed a comparable degradation range of 55.76% to 77.93% (Fig. 8D), with no statistically significant difference between them. Notably, although PCN@Thy possesses antibacterial properties, its incorporation did not hinder biodegradation. Instead, by increasing the water absorption capacity, surface roughness, and porosity of the films, it likely promoted microbial colonization and accelerated the degradation process (Brunšek et al., 2023). In conclusion, the introduction of PCN@Thy preserves the excellent eco-friendliness of the composite film, highlighting its potential as a sustainable food packaging material.

### 3.7 Preservation effects of CS/PVA/PT 1.2% on food

#### 3.7.1 Application of CS/PVA/PT 1.2% film for fillet

The appearance of the fish slices at different storage times is exhibited in Fig. 9A. Notably, the wrapping films were carefully removed under sterile conditions prior to imaging clearly visualize the surface changes of the fish slices. In the control group, the slices exhibited a loss of gloss by day 3. By day 6, they had become slimy and light brown, accompanied by a strong off-odor. Slices in the CS/PVA group showed slight darkening on day 3 and began to appear slimy on day 9. In contrast, the CS/PVA/PT group retained a desirable color and overall appearance even on day 9. The TVC in food is closely associated with its quality and serves as a critical indicator of freshness. Generally, an increase in TVC is indicative of food quality deterioration. The acceptability threshold of TVC for fresh fish is 6.0 lg CFU/g (Lan et al., 2022). While refrigeration slows down spoilage, it fails to completely arrest microbial proliferation. As manifested in Fig. 9B, the initial TVC of the fresh fish was 4.49 lg CFU/g. By day 6, the TVC in the control and CS/PVA groups had reached to 7.08 and 6.80 lg CFU/g, respectively, both exceeding the safety limit. However, the CS/PVA/PT group effectively delayed microbial growth, maintaining a TVC of 5.92 lg CFU/g on day 6, and only exceeded the limit on day 9. Therefore, these results indicate that the CS/PVA/PT film can effectively inhibit bacterial growth and extend the shelf life of fish.

**Fig. 9:**
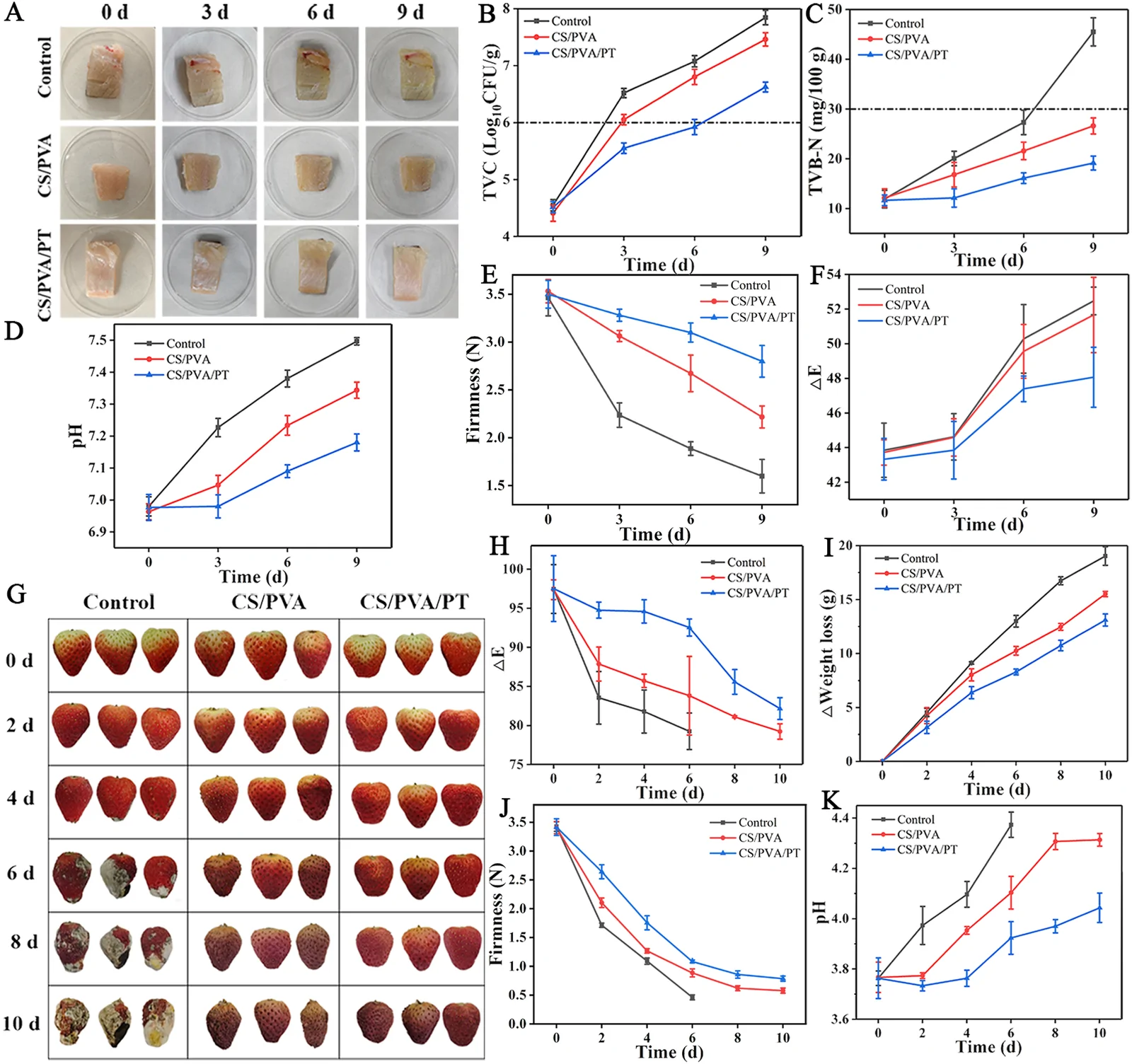
(A) Appearance, (B) TVC, (C) TVB-N value, (D) pH value, (E) hardness, and (F) ΔE of fillet in different treatment groups during storage. (G) Appearance, (H) ΔE, (I) Δweight loss, (J) hardness, and (K) pH of strawberry in different treatment groups during storage.

During storage, endogenous proteases and microbial metabolism degrade proteins into amines, ammonia, and other volatile nitrogenous compounds (Ehsani et al., 2020). Consequently, TVB-N is widely regarded as a key indicator for evaluating seafood freshness, with a safety threshold set at 30 mg/100 g (X. Wang et al., 2025b). The TVB-N evolution is depicted in Fig. 9C. The control group exhibited a rapid accumulation of TVB-N, breaching the 30 mg/100 g threshold by day 9. While the CS/PVA group marginally maintained TVB-N below the limit (26.60 mg/100 g on day 9), the CS/PVA/PT group demonstrated superior suppression, limiting TVB-N to a mere 19.13 mg/100 g. Given that TVB-N accumulation is a direct consequence of microbial proliferation (Li et al., 2020), the significantly lower TVB-N levels in the CS/PVA/PT group further corroborate the film's exceptional ability to retard protein decomposition and maintain fish freshness.

Furthermore, the pH variations of fish samples during storage are depicted in Fig. 9D. The pH values across all groups showed a progressive increase. In contrast to the rapid pH elevation in the control group (reaching 7.50 on day 9), the CS/PVA group exhibited a moderate increase, while the CS/PVA/PT group maintained the slowest rate of increase, stabilizing at 7.18. Consistent with TVB-N results, this pH rise is primarily driven by microbial proliferation and endogenous protease activity, which generate volatile basic nitrogenous compounds (Tan et al., 2024). Therefore, the delayed accumulation of these alkaline substances by the CS/PVA/PT film directly accounts for its highly effective pH stabilization.

Post-mortem proteolysis and microbial degradation disrupt the myofibrillar structure and reduce water-holding capacity, ultimately leading to a significant decline in fish hardness (Xiong et al., 2024). As illustrated in Fig. 9E, the hardness of all three groups exhibited a downward trend over the 9-day storage period at 4 °C. Specifically, the hardness of the control group decreased from 3.46 N to 1.60 N (a 54% reduction), and the CS/PVA group decreased from 3.53 N to 2.22 N (a 37% reduction). In contrast, the CS/PVA/PT composite film group showed a significantly smaller reduction of only 20% (from 3.50 N to 2.80 N). This result indicates that the film effectively mitigated the softening process, which can be attributed to its synergistic antibacterial activity, favorable barrier properties, and antioxidant capacity.

Fish color is a critical visual indicator of freshness. While fresh fish typically exhibits a bright and natural coloration, prolonged storage leads to microbial proliferation and protein decomposition, causing the flesh to gradually turn grayish-brown (Babic Milijasevic et al., 2023). After 9 days, the control group showed the most severe color deterioration, primarily driven by lipid oxidation, myoglobin oxidation, and microbial activity (Fig. 9F). In contrast, the CS/PVA/PT group maintained a relatively stable color, attributable to the film's robust antibacterial and antioxidant capabilities. To comprehensively validate the practical feasibility of this active packaging system, the migration risk of Zr was assessed using ICP-MS after 9 days of contact. Although China's National Food Safety Standard (GB 2762-2022) has not established a limit for Zr, the European Directorate for the Quality of Medicines (EDQM) issued a reference standard in 2024 suggesting a Specific Release Limit (SRL) of 2 mg/kg for Zr in food contact materials. The Zr contents detected in this study on day 0 and day 9 were 0.006 mg/kg and 0.042 mg/kg, respectively. The migration Zr was calculated to be 0.036 mg/kg. This value is negligible and well below the aforementioned reference limit, confirming the biosafety of the composite film. Overall, compared to the control group and pure CS/PVA film, the CS/PVA/PT film demonstrated superior performance in extending the shelf life of sea bass by at least 3 days.

#### 3.7.2 Application of CS/PVA/PT 1.2% film for strawberry

The study also selected strawberries, which are rich in nutrients but highly perishable, as a model to evaluate the preservation performance of packaging materials. As exhibited in Fig. 9G, the strawberries in the control group developed mold on day 4 and had severely rotted by day 6. In contrast, mold spots in the film-treated groups was significantly delayed, and color retention was notably improved. Specifically, strawberries wrapped with the CS/PVA film began to wrinkle and darken by day 6, with small mold spots appearing on day 8; whereas those in the CS/PVA/PT 1.2% group maintained a good appearance on day 8, showed only slight wrinkling, and remained free of mold spots until day 10. Consistent with the visual observations, the color difference analysis revealed that the control group exhibited the most significant color change, followed by the CS/PVA group, while the CS/PVA/PT 1.2% group showed the least change (Fig. 9H). This preservation efficacy can be explained as follows: the film acted as a physical barrier to prevent external contamination, while the chitosan (CS) in the matrix exerted an inherent antibacterial effect. Notably, for the CS/PVA/PT 1.2% film, the continuous release of Thy coupled with and the ROS generated under visible light irradiation, achieving a potent antimicrobial effect that significantly extended the shelf life of the strawberries (H. Wang et al., 2025a).

To comprehensively evaluate the preservative efficacy of the CS/PVA/PT film on strawberries, weight loss and hardness were also assessed. Weight loss in fruits during storage primarily stems from respiration and transpiration (H. Wang et al., 2025b), and is therefore closely dependent on the barrier properties of the packaging. As shown in Fig. 9I, the control group exhibited the most significant weight loss over time, whereas the film-wrapped groups maintained relatively lower losses. This preservation effect occurred because the films acted as a physical barrier, effectively retarding water vapor evaporation and reducing substrate consumption from respiration (Pinzon et al., 2020). Notably, the CS/PVA/PT 1.2% group exhibited the lowest weight loss, attributed to the incorporation of PCN@Thy further enhanced the overall barrier performance of the composite film.

Additionally, all groups exhibited a gradual decline in firmness during storage (Fig. 9J), a deterioration driven by water loss and cell wall degradation (Zhao et al., 2019). The unpackaged fruits were directly exposed to an aerobic environment, which led to a significant increase in the activity of cell wall degrading enzymes (e.g., pectinases). This accelerated the degradation of cell wall components and subsequently caused fruit softening (Daglio et al., 2024). In contrast, the slower softening of film-wrapped strawberries, particularly in the CS/PVA/PT group, was primarily attributed to the mitigation of environmental stresses. Specifically, the barrier properties effectively retarded water loss and reduced oxygen availability to suppress respiration (Gammage and Marangoni, 2025). Moreover, the antimicrobial and antioxidant capabilities of the CS/PVA/PT film played a critical role by preventing exogenous microbial invasion and scavenging endogenous free radicals, thereby collectively safeguarding cellular structural integrity (Liu et al., 2023).

Furthermore, strawberries wrapped with CS/PVA/PT exhibited the lowest change in pulp pH over the 10-day period (Fig. 9K). This stability can be attributed to two main factors: First, the outstanding barrier properties of the film regulated the internal gas composition, inhibiting respiration metabolism and thereby delaying the consumption of organic acids, which serve as primary substrates for respiration (Pinzon et al., 2020); Second, the excellent antibacterial properties effectively suppressed the proliferation of spoilage microorganisms (as previously shown in Fig. 7A), minimizing the microbial depletion of organic acids and the subsequent production of alkaline metabolites (Tezotto-Uliana et al., 2014). Collectively, these obtained results demonstrate that the CS/PVA/PT film is highly effective in maintaining the freshness of strawberries.

## 4 Conclusion

In conclusion, the synergistic encapsulation of thymol within porphyrin-based porous frameworks (PCN-224) integrated into a CS/PVA matrix successfully yields a multifunctional active film with enhanced preservation capabilities. The engineered synergy between the sustained release of thymol and light-triggered ROS generation collectively endows the film with robust antibacterial, antifungal, and antioxidant properties. Practical evaluations confirm its significant value in food preservation: it effectively retarded fish spoilage by controlling TVB-N, TVC, and pH, and maintained strawberry quality by suppressing microbial growth and physical deterioration. Safety was also verified by the negligible Zr migration. While the current system relies on light availability and requires further long-term nano-toxicological evaluation, this work provides a paradigm for designing synergistic porphyrin-framework-based active packaging. Future efforts will focus on enhancing photoconversion efficiency for low-light environments and comprehensive safety validation to accelerate commercial translation.

## Author contributions

The manuscript was written through contributions of all authors. All authors have given approval to the final version of the manuscript.

## CRediT authorship contribution statement

Huajuan Wang: Conceptualization, Funding acquisition, Project administration, Writing – review & editing. Mengni Zhou: Investigation, Methodology, Writing – original draft. Yeting Xu: Data curation, Formal analysis, Methodology. Yan Huang: Formal analysis, Methodology. Liang Ke: Formal analysis, Methodology. Wei Xu: Data curation, Formal analysis. Yang Yi: Investigation, Visualization. Min Zhou: Conceptualization, Funding acquisition, Project administration.

## Declaration of competing interest

The authors declare that they have no known competing financial interests or personal relationships that could have appeared to influence the work reported in this paper.
