# Supplementary material for: Synergistic encapsulation of thymol in porphyrin-based porous frameworks enhances the multifunctionality of chitosan/polyvinyl alcohol films for food preservation
Source: Curr Res Food Sci. 2026 Oct 20;13:101605. doi: 10.1016/j.crfs.2026.101605 (PMC13718868; doi:10.1016/j.crfs.2026.101605)
Supplement: Supplementary file 1 [file mmc1.docx]

**Supporting Information**

**Synergistic Encapsulation of Thymol in Porphyrin-Based Porous Frameworks Enhances the Multifunctionality of Chitosan/Polyvinyl Alcohol Films for Food Preservation**

Huajuan Wang^a^*, Mengni Zhou^a^, Yeting Xu^a^, Yan Huang^b^, Liang Ke^cd^, Wei Xu^a^, Yang Yi^a^, Min Zhou^a^*

a. Hubei Key Laboratory for Processing and Transformation of Agricultural Products, College of Food Science and Engineering, Wuhan Polytechnic University, Wuhan, 430023, China.

b. Huangshi Central Hospital, Huangshi, 435000, China.

c. Hubei Huangshan Industry Group Company Limited, Xiantao, 433022, China.

d. Hubei Huangshan Industry Technology Research Institute Company Limited, Xiantao, 433022, China.

* Corresponding author.

**1. Materials and methods**

**1.1** **Regents and materials**

N, N-dimethylformamide (DMF), Dimethyl Sulfoxide (DMSO), and tetrakis(4-carboxyphenyl) porphyrin (TCPP, 97 wt%) were obtained from Shanghai Yuanye Bio-Technology Co., Ltd. SYTO 9, 2’,7’-dichlorodihydrofluorescein diacetate (DCFH-DA), crystal violet, BCA protein assay kit, propidium iodide (PI), and 3-(4,5-Dimethylthiazol-2-yl)-2,5-diphenyltetrazolium bromide (MTT) were all acquired from Beyotime Institute of Biotechnology. In addition, phosphate buffered saline (PBS) and ethanol (99.7 wt%) were got from Shanghai Chemical Reagent Co., Ltd.

**1.2 Characterization of PCN@Thy nanoparticles**

The morphology of PCN@Thy was observed using transmission electron microscopy (TEM, JEM-2010, Japan) and scanning electron microscopy (SEM, SU8100, Japan). The hydrodynamic particle size and zeta potential were measured with a Malvern Zetasizer (ZS90). Furthermore, Fourier transform infrared (FTIR) spectroscopy, ultraviolet–visible (UV-vis) spectroscopy, and X-ray diffraction (XRD) were employed to investigate the structural properties of PCN@Thy. The hydrophilicity of the films was evaluated through water contact angle (WCA) measurements.

**1.3 Live/dead staining assay**

Bacterial suspensions (10^5^ CFU/mL) were divided into four treatment groups: PBS (control), free Thy at an equivalent concentration, an equivalent amount of PCN-224 plus visible light irradiation (633 nm, 10 min), and PCN@Thy (120 μg/mL) plus visible light irradiation (633 nm, 10 min). Following treatment, the suspensions were incubated for an additional 3 h. Subsequently, the bacteria were stained with PI and SYTO 9 in the dark for 30 min. After washing with PBS, the fluorescence signals of the bacterial cells were visualized using a confocal laser scanning microscope (CLSM, FV1200, Japan).

**1.4 Detection of intracellular ROS**

Bacterial suspensions were treated with PBS (control), CS/PVA, and CS/PVA/PT films under visible light irradiation for 10 min, and then incubated at 37 °C for 3 h. After washing with PBS, the bacteria were incubated with DCFH-DA probe with DCFH-DA (10 μM) in the dark for 20 min. Finally, the fluorescence signals of the bacterial samples were observed using a CLSM.

**1.5 Leakage of intracellular proteins**

Bacterial suspension (10⁸ CFU/mL, 100 μL) were treated as follows: PBS (Control), CS/PVA film, and CS/PVA/PT film (containing 1.2% PCN@Thy, followed by 10 min of visible light irradiation). After incubation for 3 h, the supernatants were collected, and the concentration of leaked proteins was quantified using a BCA protein assay kit.

**1.6 Antibiofilm test**

Inhibition of biofilm formation (H. Wang et al., 2025): Bacterial suspensions (10⁵ CFU/mL) were co-incubated with different concentrations of PCN@Thy (0, 30, 60, 90 and 120 μg/mL). The mixtures were exposed to visible light for 10 min, followed by a 3-h incubation. Subsequently, the cultures were allowed to stand for 48 h to facilitate biofilm development. The resulting biofilms were stained with crystal violet, gently washed with PBS, and dissolved in ethanol. Finally, biofilm formation was quantified by measuring the absorbance of the solution at 590 nm (OD590).

Scavenging effect on mature biofilms (H. Wang et al., 2025): Bacterial suspensions (10⁵ CFU/mL) were inoculated into 96-well plates containing TSB medium and statically cultured for 48 h to form mature biofilms, with fresh TSB medium replaced every 24 h. Subsequently, different concentrations of PCN@Thy (0, 30, 60, 90 and 120 μg/mL) were added to the wells and exposed to visible light for 10 min. After an additional 24 h of incubation, planktonic bacteria were removed by washing with PBS, and the adherent biofilms were stained with crystal violet solution. Finally, the crystal violet was dissolved in ethanol, and the OD₅₉₀ of the solution was measured.

**1.7 Physical and optical properties of the films**

The thickness of the films was measured at five randomly selected positions using a handheld micrometer, and the average was calculated. The surface color parameters (L*, a*, b*) were determined using a colorimeter (L* = 94.28, a* = -0.08, b* = 0.62) (WN700D, China) (Woźniak et al., 2024). Additionally, the optical transmittance of the composite films at 280 nm (T280) and 660 nm (T660) was recorded using a UV-Vis spectrophotometer (Cai et al., 2025). The transmittance (T, %) was calculated using the equation:

T (%) = 10 -A × 100%, where A is the absorbance value.

**1.8 Water vapor permeability (WVP)**

The WVP of the films was determined according to previous method with slightly modification (H. Wang et al., 2025). Briefly, a weighing bottle containing anhydrous calcium chloride (0% RH) was sealed with the film sample using an elastic band. The sealed bottle was then placed in a desiccator containing distilled water (100% RH) and maintained at 25 °C. Weighing the total weight of the weighing bottle every 2 h until a steady state of water vapor transmission was reached (typically after 12 h). The WVP was calculated according to the following formula:

WVP = (∆m×X) / (∆t×A×∆P)

where ∆m is the change in mass (g), ∆t is the change in time (h), A is the effective permeation area (m²), X is the average film thickness (m), and ∆P is the water vapor pressure difference across the film (3169 Pa).

**1.9 Oxygen permeability (OP)**

An oxygen scavenger (composed of iron powder, activated carbon, and NaCl) was placed inside a weighing bottle. The bottle mouth was then sealed with the composite film, and the assembly was transferred to a desiccator containing a saturated BaCl₂ solution for 48 h(H. Wang et al., 2025). The OP was calculated using the following formula:

OP = (∆m×X) / (A×t)

where Δm is the weight gain of the bottle (g), X is the film thickness (m), t is the testing time (h), and A is the effective sealing area of the film (m²).

**1.10 Cytotoxicity evaluation**

Film samples (1 g) were immersed in 10 mL of DMEM medium containing 10% fetal bovine serum (FBS) for 24 h to prepare the extract (X. Wang et al., 2025). The resulting sterile extract was then collected for further use.

MTT assay: Caco-2 cells were seeded into 96-well plates and cultured to a density of 1 ×10^4^ cells/mL and exposed to the extracts for 24 h. Following PBS washing, the cells were recovered in fresh DMEM medium (10% FBS) for 8 h. Then, 20 μL of MTT solution was added, and the plate was incubated for 4h. After discarding the supernatant, 150 μL of DMSO was added to dissolve the formazan crystals. Finally, the absorbance at 490 nm was measured to determine cell viability, with cells cultured in pure DMEM medium serving as the control.

Hemolysis Assay: Briefly, 400 μL of film extract was mixed with 100 μL of red blood cell (RBC) suspension in a centrifuge tube. The mixture was incubated at 37°C for 4 h. After centrifugation (3000 rpm, 5 min), the supernatant was collected, and its absorbance was measured at 540 nm. RBCs treated with distilled and physiological saline (0.9% NaCl) served as the positive and negative controls, respectively.

**1.11 Application of fish preservation**

pH measurement: Fish samples (3 g) were homogenized in 27 mL of ultrapure water for 10 min. Then, the pH value of the resulting supernatant was then measured using a calibrated pH meter.

Total viable count (TVC): Fish samples (3 g) were homogenized in 27 mL of sterile PBS for 3 min. Following serial dilution, appropriate dilutions of the supernatant were mixed with plate count agar (PCA) and poured into petri dishes. The plates were incubated at 30 °C for 48 h to determine the TVC.

Firmness: Fish samples were cut into cubes (1.0 cm × 1.0 cm × 1.0 cm) for texture analysis using a texture profile analyzer. The test parameters were set to a compression speed of 1.0 mm/s, a trigger force of 5 g, and a compression ratio of 60%.

Color difference (Δ𝐸): The color parameters of the samples were measured using a colorimeter (calibrated with a standard white tile: L*=94.28，a*=-0.08，b*=0.62). At least three different positions on each sample were measured and averaged. The Δ𝐸 was calculated by comparing the values to those of the initial samples (day 0).

**1.12 Application of strawberry preservation**

Firmness: The firmness of the strawberries was measured at four different positions around the equatorial region using a 5 mm diameter spherical probe, and the average value was calculated. The texture profile analyzer was set with the following parameters: test speed of 1 mm/s, trigger force of 5 g, deformation of 30%, and a 5 s interval between compressions. The maximum peak force (N) recorded during the test was used as the firmness indicator.

pH: Strawberry pulp (10 g) was homogenized with 100 mL of ultrapure water using a high-speed homogenizer. The resulting homogenate was filtered, and the pH of the filtrate was directly measured using a digital pH meter.

Δ𝐸: The surface color of the strawberries at the equatorial region was measured using a colorimeter (calibrated with a standard white tile: L*=94.28, a*=-0.08, b*=0.62). The L*, a*, and b* values were recorded, and the ΔE was calculated relative to the initial values (day 0).

**2. Results and Discussion**

Oxidative stress caused by ROS is a primary antibacterial mechanism of PCN-based materials. To visually confirm the intracellular ROS levels, bacterial cells were stained with a fluorescent probe. As shown in **Figure S1**, the green fluorescence intensity corresponds to the ROS concentration. No fluorescence was observed in the control group, whereas the CS/PVA-treated group displayed only weak background fluorescence. In contrast, the CS/PVA/PT-treated group exhibited a strong green fluorescence signal under visible light irradiation, indicating the efficient generation of ROS by the composite film.

**Figure S1.** The fluorescence images of ROS within bacterial cells under different treatment conditions.

The disruption of the cell membrane leads to the release of intracellular components. Here, the leakage of extracellular proteins from *P. aeruginosa* was used as an indicator of membrane damage. As depicted in **Figure S2**, no significant protein leakage was detected in the control group, indicating an intact cell membrane. Treatment with the CS/PVA film caused a slight increase in protein leakage, while the CS/PVA/PT group exhibited a significantly higher amount of leakage. This quantitatively confirms that the composite film induces more severe physical damage to the bacterial membrane.

**Figure S2.** The leakage of proteins from *P. aeruginosa* after being treated with different films.

Beyond structural damage, functional impairment of the cell membrane was evaluated by measuring membrane potential. Damage to the cell membrane can lead to alterations in membrane potential, and these changes can be detected by observing the changes in the fluorescence intensity of Rhodamine 123. As depicted in **Figure S3**, compared with the control group, the fluorescence intensity of bacteria treated with PCN@Thy was significantly (*p* < 0.05) attenuated. Notably, this decrease exhibited a concentration-dependent manner, where fluorescence intensity gradually reduced as the concentration of PCN@Thy increased. This result provides direct evidence of membrane depolarization and dysfunction, corroborating the membrane integrity damage observed in the leakage assays.

**Figure S3.** The membrane potential of bacteria treated with different concentrations of PCN@Thy.

**References**

Wang, H., Shi, T., Ma, J., Meng, S., Wei, Z., Sun, Y., Wang, H., & Zhou, M. (2025). Chitosan-based nanocomposite films incorporated with AgNPs/porphyrinic MOFs for killing pathogenic bacteria. International Journal of Biological Macromolecules, 295, 139584.

Woźniak, M., Młodziejewska, J., Stefanowska, K., Mrówczyńska, L., Sip, A., Dobrucka, R., & Ratajczak, I. (2024). Chitosan-based films with essential oil components for food packaging. Coatings, 14(7), 830.

Cai, X., Chen, L., Yang, X., Wang, Y., Xu, J., Zhang, R., Ling, S., & Liu, Y. (2025). Active curcumin-loaded γ-cyclodextrin-metal organic frameworks as nano respiratory channels for reinforcing chitosan/gelatin films in strawberry preservation. Food Hydrocolloids, 159, 110656.

Wang, X., Wang, C., Zhao, J., Zhang, X., Liu, K., & Yang, J. (2025). A trilaminar edible film of nanocellulose, konjac glucomannan, and curcumin with pH-responsive and amphiphilic properties: preparation, characterization, and application. Food Chemistry, 488, 144913.

Wang, H., Wu, Y., Wang, Y., Zou, H., Wang, H., & Zhou, M. (2023). Vancomycin-Loaded Porphyrinic MOF Nanoparticles for Killing Pathogenic Bacteria. ACS Applied Nano Materials.
